# Integrated multi-omics and machine learning reveal an immunogenic cell death-related signature for prognostic stratification and therapeutic optimization in colorectal cancer

**DOI:** 10.3389/fimmu.2025.1606874

**Published:** 2025-07-16

**Authors:** Siyu Hou, Shanshan Heng, Shaozhuo Xie, Yuanchun Zhao, Jiajia Chen, Chunjiang Yu, Yuxin Lin, Xin Qi

**Affiliations:** ^1^ School of Chemistry and Life Sciences, Suzhou University of Science and Technology, Suzhou, China; ^2^ Suzhou Industrial Park Institute of Services Outsourcing, Suzhou, China; ^3^ Department of Urology, the First Affiliated Hospital of Soochow University, Suzhou, China; ^4^ Center for Systems Biology, Soochow University, Suzhou, China

**Keywords:** colorectal cancer, single-cell RNA sequencing, immunogenic cell death, macrophage, immunotherapy

## Abstract

Colorectal cancer (CRC) continues to rise in global incidence and remains a leading cause of cancer-related mortality. Immunogenic cell death (ICD) has emerged as a critical modulator of tumor microenvironment (TME) dynamics; however, its prognostic implications and therapeutic potential in CRC require systematic characterization. Through the integrative analysis of single-cell RNA sequencing and bulk transcriptomic data, 11 ICD-related genes with prognostic significance were identified in CRC. A comprehensive computational framework was then employed to evaluate 101 machine learning combinations, ultimately constructing an optimized 11-gene ICD-related signature (ICDRS) by integrating StepCox [forward] and RSF. The ICDRS exhibited strong predictive performance for overall survival in CRC patients across the training and validation datasets. Notably, the ICDRS-based nomogram achieved outstanding time-dependent AUCs (>0.90) for 1- to 3-year survival prediction. Multidimensional analysis revealed significant associations between ICDRS-derived risk score and distinct immune infiltration patterns, immunotherapy response and TME characteristics. Furthermore, a novel macrophage subtype, SPP1^+^/SLC11A1^+^, was discovered and characterized by high infiltration levels. Drug repurposing analysis indicated Olaparib as a potential therapeutic candidate for high-risk CRC patients. Therefore, this study establishes ICDRS as a promising tool for CRC prognosis and immunotherapy, with future validation studies planned to guide personalized treatment strategies.

## Introduction

Colorectal cancer (CRC) ranks as the third most common and second deadliest cancer worldwide, accounting for about 9.4% of all cancer-related deaths in 2020 (1). By 2030, the number of new CRC cases globally is projected to exceed 2.2 million, with an estimated 1.1 million deaths, posing a serious challenge to public health (2). Despite advancements in bowel cancer screening, approximately 20% of patients are diagnosed at an advanced stage, with a historically 5-year overall survival (OS) rate of about 13% for these CRC patients (3). Therefore, improving OS remains the primary objective in CRC researches due to its significant influence on patient care and treatment outcomes.

Immunogenic cell death (ICD) refers to a stress-driven form of cell death that activates anti-tumor immune response by releasing danger associated molecular patterns (DAMPs) and tumor-associated antigens from dying tumor cells (4). Especially, it can facilitate the maturation of dendritic cells and the infiltration of cytotoxic T lymphocytes, potentially reversing the tumor immunosuppressive microenvironment to enhance immunotherapy sensitivity (5, 6). Recently, it has been reported that biomarkers associated with ICD can provide valuable insights into the tumor’s behavior and the patient’s prognosis across a variety of cancer types. For example, Cai et al. (7) constructed an ICD-related risk signature that can be used for predicting prognosis and the sensitivity to immune checkpoint blockade (ICB) immunotherapy in patients with lower-grade glioma. Similarly, Lei et al. (8) identified a panel of crucial ICD-related genes that modulate the tumor immune microenvironment (TME) and the progression of CRC, highlighting their significance for CRC prognosis and therapeutic targeting. Therefore, leveraging ICD for identifying biomarkers and developing prognostic models holds the potential to significantly advance cancer treatment by improving patient outcomes.

Although ICD plays a promising role in cancer immunotherapy, yet to date, there are only a limited number of oncological therapeutic agents that can trigger ICD, mainly including Anthracyclines, Oxaliplatin, Cetuximab, and Bortezomib (9). In response to this limitation, researchers are actively focusing on developing and investigating new drugs that can effectively induce ICD. Especially, drug repurposing emerges as an efficient and cost-effective approach to identify potential therapeutic options that can induce ICD from existing drugs, offering a novel perspective for the treatment of CRC. For example, Grazia et al. (10) discovered that the anti-helmintic drug rafoxanide can inhibit proliferation and induce ICD in murine CRC cells. Moreover, immunocompetent mice immunized with dying tumor cells treated with rafoxanide had a significantly increased tumor-free survival compared with sham-operated mice, highlighting the anti-cancer vaccination effect of rafoxanide on CRC. Furthermore, Yu et al. (11) proposed a combination treatment strategy that co-delivers Oxaliplatin and rapamycin (an immunosuppressive drug used for organ transplantation) delivered by folate-modified nanoliposomes for CRC. Notably, this innovative approach effectively inhibited tumor growth and liver metastasis of CRC by inducing ICD, providing a promising strategy for CRC treatment. Therefore, exploring drugs acting as ICD inducers has significant implications in cancer treatment.

**Figure 1:**
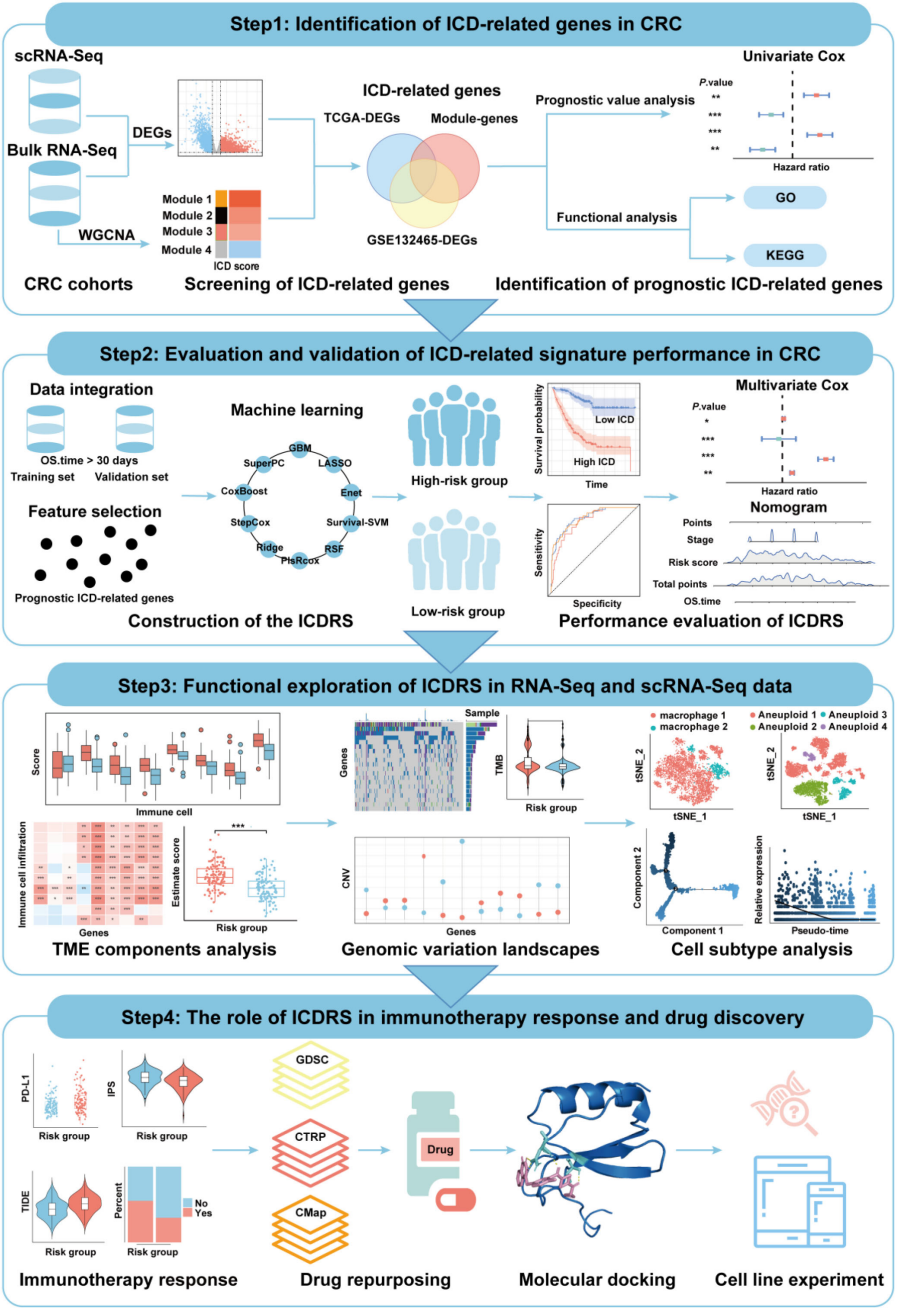
Flowchart of the present study.

Recently, advances of single-cell transcriptomics have significantly enhanced our understanding of cellular heterogeneity and dynamic interactions within the TME, which is crucial for identifying new therapeutic targets and biomarkers. In the present study, we aim to develop and validate a novel ICD-related prognostic signature by integrating single-cell RNA-Seq and bulk RNA-Seq data based on a computational machine learning framework containing 101 combination algorithms. The role of the established signature in CRC was explored from multiple perspectives including molecular function, interactions within TME and immunotherapy response. Besides, potential drugs for CRC were repositioned based on the signature-related protein-protein interaction network. Therefore, this study not only advances our understanding of ICD in CRC, but also offers promising avenues for therapeutic intervention and improved patient outcomes (**Figure 1**).

## Materials and methods

### Data collection and preparation

To explore ICD-related genes with prognostic value in CRC at the single-cell and bulk transcriptome levels, the present study first downloaded the CRC single-cell dataset GSE132465 from the Gene Expression Omnibus database (GEO, https://www.ncbi.nlm.nih.gov/geo/), which comprises 23 primary CRC tumor samples and 10 matched normal mucosa samples. Besides, transcriptomic and clinical data of 517 COAD samples and 98 READ samples were obtained from The Cancer Genome Atlas (TCGA, https://portal.gdc.cancer.gov/). The RNA-Seq raw read counts were transformed into transcripts per kilobase million (TPM) for further analysis. Furthermore, to develop and validate an ICD-related prognostic signature, microarray datasets including GSE17538 (n=232), GSE17537 (n=55), GSE17536 (n=177), GSE38832 (n=122), and GSE29621 (n=65) were obtained from GEO database. To ensure the stability and reliability of prognostic signature, CRC samples lacking survival and TNM stage information, as well as those with OS or disease-free survival (DFS) less than 30 days were excluded. To validate the expression levels of the genes in CRC paired samples, the GSE44861 (n=55) and GSE87211 (n=260) datasets were utilized.

### Single-cell RNA-Seq analysis

To identify genes involved in the ICD process at the single-cell transcriptome level, the “Seurat” R package (version 5.1.0) (12) was used to analyze the scRNA-Seq dataset GSE132465. First, to filter out low-quality cells and retain only biologically meaningful data, quality control was applied based on the following thresholds criteria: cells were retained if they had less than 20% mitochondrial gene content and expressed at least 1,000 genes (13). Additionally, only genes expressed in at least three cells, with an expression range between 200 and 6,000, were included. Next, the top 1,000 highly variable genes were selected to capture meaningful biological variability while reduce noise and computational complexity. Batch effects across different samples were removed using the “Harmony” R package. The “FindNeighbors” and “FindClusters” functions in the “Seurat” R package were then employed to identify cell clusters with a resolution of 0.5. For cluster visualization, the “t-SNE” (14) dimensionality reduction method was applied. Finally, the following canonical biomarkers were used for cluster annotation of specific cell types: epithelial cells (CDH1, EPCAM, and CLDN4), fibroblasts (COL1A1, COL1A2, and DDR2), macrophages (CD68, CD14, and CD163), B cells (MS4A1, CD19, and BANK1), plasma cells (TNFRSF17, MZB1, and DERL3), endothelial cells (CLDN5, VWF, and KDR) and T cells (CD3D, CD3E, and CD3G). A total of 34 ICD-related genes were collected from previously published literature to explore the prognostic role of ICD in CRC (15, 16) (**Supplementary Table S1**). The single-cell ICD score was calculated using the “AddModuleScore” function from the “Seurat” package, which quantifies the expression activity of the ICD-related gene set at the single-cell level. Cells were subsequently divided into high-ICD activity and low-ICD activity groups based on median activity scores. Differentially expressed genes (DEGs) between these groups were identified using the “FindMarkers” function in the “Seurat” R package, with selection criteria of |log_2_Fold change (FC)|>0.585 and *P*.adj<0.05.

### Weighted gene co-expression network analysis based on bulk RNA-seq data

To identify the gene module with the highest correlation to ICD in TCGA-CRC dataset, the WGCNA analysis was conducted using the R package “WGCNA” (version 1.72.5) (17), in which ICD score computed via the ssGSEA algorithm were employed as phenotypic data. During the analysis, an optimal soft threshold was determined with a power value of 8. The weighted adjacency matrix was then transformed into a topological overlap matrix (TOM), and the dissimilarity (dissTOM) was computed. When constructing the co-expression network, the minimum module gene number was set to 300, and the MEDissThres was adjusted to 0.3. Pearson correlation coefficient and Student’s t-test were used to identify the module with the highest correlation between module eigengenes (MEs) and ICD score. Further considering the expression level changes at the individual gene level, the “edgeR” R package (18) was employed to identify DEGs between CRC samples and normal samples in the TCGA-CRC dataset under the same criteria (|log_2_FC|>0.585 and *P*.adj<0.05) (19). Finally, the ICD-related genes in CRC were identified through a Venn analysis, intersecting DEGs from the high- and low-ICD activity groups in the GSE13246 dataset, DEGs from tumor and normal samples in the TCGA-CRC dataset, and genes from the ICD-related module identified by the WGCNA analysis in the TCGA-CRC dataset. ICD-related genes with potential prognostic significance in CRC were further identified though a univariate Cox regression analysis.

### Functional enrichment analysis

To investigate the function of the ICD-related genes in CRC, the “clusterProfiler” R package (version 4.8.3) (20) was used to conduct gene ontology (GO) and Kyoto Encyclopedia of Genes and Genomes (KEGG) enrichment analyses. The *P* values were adjusted using the Benjamini-Hochberg (BH) correction method, and significantly enriched terms were selected based on the criterion of *P*.adj < 0.05.

### Construction and validation of the immunogenic cell death-related signature

To construct an accurate and robust immune-related cell death signature (ICDRS) for CRC prognosis, an expanded number of datasets were selected, where the GSE17538 was served as the training set, and GSE17537, GSE17536, GSE29621, GSE38832, and TCGA-CRC were chosen for external validation. Given the intrinsic heterogeneity of CRC and the complexity of ICD mechanisms, reliance on a single machine learning algorithm may introduce bias or fail to capture the complete prognostic landscape. To enhance model robustness and generalizability, an integrated computational framework incorporating ten state-of-the-art machine learning algorithms, including Cox Boost, Elastic Net (Enet), Gradient Boosting Machine (GBM), least absolute shrinkage and selection operator (LASSO), partial least squares regression for Cox (plsRcox), random survival forest (RSF), Ridge, stepwise Cox, supervised principal components (SuperPC), and survival support vector machine (survival-SVM), was implemented. Each feature selection method was systematically paired with each modeling algorithm, resulting in a total of 101 algorithmic combinations. The C-index values of the signatures developed by the combined algorithms were calculated on both training and validation sets, respectively. The predictive performance of the model was then ranked based on the average C-index, and the algorithm combination with the highest average C-index was selected as the optimal strategy to construct the ICDRS. The risk score of each CRC patient could be quantified using the optimal algorithm combination, and CRC patients were further divided into high-risk and low-risk groups based on the median of the risk score.

To evaluate the discriminative ability of ICDRS, the “survminer” R package was used to plot the Kaplan-Meier survival curves of the high-risk and low-risk groups, assessing whether there were significant differences between the two groups in terms of OS or DFS (log-rank test, *P*<0.05). Additionally, the “timeROC” R package was used to perform receiver operating characteristic (ROC) curve analysis, evaluating the sensitivity and specificity of ICDRS in predicting OS or DFS in CRC patients.

Moreover, to measure the prognostic value of ICDRS, sixty reported prognostic signatures for CRC, associated with various biological characteristics, such as immune response, autophagy, and ferroptosis, were collected from the PubMed (https://pubmed.ncbi.nlm.nih.gov/) (**Supplementary Table S2**). The “compareC” R package was used to compare the performance of ICDRS with other signatures. Subsequently, the multivariate Cox regression analysis was conducted to evaluate the independence of ICDRS and clinicopathological factors in predicting CRC prognosis.

### Development of an ICDRS-based nomogram for monitoring CRC prognosis

A novel nomogram was developed by incorporating ICDRS with clinical characteristics for personalized prediction of the OS of CRC patients. The predictive ability of the nomogram was measured by the area under the curve (AUC) of the ROC curve. Then, a calibration curve analysis was conducted to assess its performance by comparing predicted probabilities with actual observed probabilities. Moreover, the “rmda” R package was used to draw DCA curves and to evaluate the clinical utility of ICDRS by quantifying the net benefit at different threshold probabilities.

### Comparison of immune cell infiltration levels between high- and low-risk groups

To investigate the relationship between ICDRS and immune cell infiltration levels in the TME of CRC, ssGSEA algorithm was used to calculate and compare the enrichment scores of infiltrating immune cells and immune-related functions. The algorithm calculated 28 different types of tumor-infiltrating immune cell phenotypes, including CD8^+^ T cells, dendritic cells, macrophages, and regulatory T cells (21) (**Supplementary Table S3**). In addition, the relationship between immune cell populations and the ICDRS-derived risk scores was assessed using the Pearson correlation coefficient via the “corrplot” R package. Additionally, the ESTIMATE algorithm (22) was employed to calculate immune scores, ESTIMATE scores, tumor purity score and stromal scores between the high-risk and low-risk groups, and the Wilcoxon test was used to determine whether significant differences in scores between the two groups were observed.

### Somatic mutation and genomic variation analysis

To determine the accumulated mutation burden of CRC cells in the high-risk and low-risk groups, tumor mutation burden (TMB) analysis was performed. The mutation annotation format (MAF) was downloaded from the TCGA database using the “TCGAmutations” R package. The “maftools” R package (version 2.16.0) (23) was then employed to identify somatic mutations in the high-risk and low-risk CRC populations. The TMB scores were calculated for each CRC patient in the TCGA-CRC cohort using the “tmb” function, and the Wilcoxon test was used to evaluate statistical significance. In addition, synonymous and nonsynonymous mutations were analyzed using the “somaticInteractions” function to investigate the correlation of the top 20 mutation genes between the high-risk and low-risk groups.

In addition, copy number variations (CNV) analysis was performed on the 11 genes that make up ICDRS to better understand changes in their expression levels during the mutation process. First, the CNV data for CRCs was downloaded from the TCGA database using the “TCGAbiolinks” R package (version 2.28.4) (24). The data was then formatted for compatibility with the GISTIC module on the GenePattern website (https://cloud.genepattern.org/gp), enabling the online analysis of CNV for various genes.

### Identification of malignant cell types

To accurately distinguish somatic cells with chromosomal copy number alterations, “copykat” and “infercnv” R packages were employed to identify malignant cells in 23 CRC patients from the GSE132465 dataset. The “copykat” R package was used to infer the chromosome ploidy of single-cell transcriptome data in CRC, enabling the classification of cells as diploid (normal) or aneuploid (malignant). Next, the “infercnv” R package was used to compare gene expression levels across different cell types to determine the presence of chromosomal amplifications or deletions. A threshold of 0.1 was used to distinguish true biological changes in gene expression from background noise, thereby ensuring the identification of uniquely significant CNVs.

### Pseudo-temporal analysis

A pseudo-time analysis was performed to investigate whether the expression levels of the 11 genes comprising ICDRS changed during the differentiation process of malignant cells and macrophages. The “monocle” R package (version 2.28.0) was utilized to visualize the differentiation trajectories of malignant cell and macrophage subpopulations, as well as to analyze the ICD-related gene expression levels within each subpopulation.

### Cell-cell communication analysis

To further analyze the intercellular interactions between aneuploid cells, SPP1^+^/SCL11A1^+^ macrophages and SPP1^-^/SCL11A1^-^ macrophages, and other cells in the TME, the “CellChat” R package (version 1.6.1) was applied to conduct cell communication analysis on scRNA-Seq data from 23 CRC patients in the GSE132465 dataset.

### Assessment of immunotherapeutic responses

To evaluate the response of individuals to immunotherapy in high- and low-risk groups, the “ggplot2” R package was used to plot the expression levels of immune checkpoint genes in the high-risk and low-risk groups in the GSE17538 dataset, and Student’s t-test was used to analyze the statistical significance between the two groups. Subsequently, the “IOBR” R package (version 0.99.9) (25) was used to predict the immunophenoscore (IPS) of patients in the high-risk and low-risk groups. Furthermore, the Tumor Immune Dysfunction and Exclusion (TIDE) platform (http://tide.dfci.harvard.edu/) (26) was used to analyze the TIDE-related scores (stromal score, immune score, ESTIMATE score, and tumor purity score) for high- risk and low-risk groups. The Wilcoxon test was employed to analyze the significant differences in TIDE-related scores between the high- and low-risk groups, with a significance threshold of *P*<0.05.

### Drug repositioning analysis

To identify potential drugs for treating CRC and to evaluate the response of clinical patients to candidate drugs, a comprehensive screening was conducted in three databases: GDSC (https://www.cancerrxgene.org/), CTRP (https://portals.broadinstitute.org/ctrp/), and CMap (https://clue.io/about). Drug sensitivity was inferred using the half-maximal inhibitory concentration (IC50) of anticancer drugs. In detail, the “oncoPredict” R package (version 1.2) (27) was first used to screen candidate drugs with high drug sensitivity in the GDSC and CTRP databases. Subsequently, DEGs identified by the “limma” R package between the high-risk and low-risk groups in GSE17538 were submitted into the CMap database to identify small molecule drugs. Those with scores below -60 were selected as candidate drugs. Finally, the candidate drugs that are present in all three aforementioned databases were selected as potential drugs for CRC treatment. The “corrplot” R package was used to explore the correlation between ICDRS and drug sensitivity IC50 using Pearson correlation analysis. Wilcoxon test was used to compare the differences in drug sensitivity between the high-risk and low-risk groups in the GSE17538 dataset. In addition, this study utilized ADMETlab 3.0 (https://admetlab3.scbdd.com/server/screening) (28) and SwissADME databases (http://www.swissadme.ch/) (29) to analyze the ADMET properties of the candidate drugs.

### Protein–protein interaction network construction and molecular docking

Given the important role of protein-protein interaction (PPI) networks in identifying drug targets, this study constructed a PPI network to predict the targets of repositioned CRC drugs. Firstly, DEGs between high- and low-risk groups in the GSE17538 dataset was identified using the “limma” R package. Then, these DEGs were uploaded to the STRING database (https://cn.string-db.org/) to construct a PPI network with a confidence level of 0.4. Subsequently, the constructed PPI network was visualized and subjected to topological analysis using Cytoscape software (3.7.0) (30), and hub genes were selected using the filtering criterion of the top 5% degree values.

To further analyze the interactions between the identified drugs and hub proteins, the 3D structure of the candidate drugs was obtained from the PubChem database (https://pubchem.ncbi.nlm.nih.gov/). The 3D structures of the proteins MMP9 (PDB ID: 1l6j), CCL2 (PDB ID: 6ctw), and CXCL8 (PDB ID: 6wzm), were downloaded from the PDB database (https://www.rcsb.org/). The self-ligands and water molecules of the proteins were removed using Pymol software (2.4.0), and the 3D structures of the protein and candidate drug were then hydrogenated and docked using AutoDock software (4.2.6) (31). Finally, the results of the molecular docking were analyzed and visualized using Pymol software.

### Cell lines culture

Human CRC cell lines (HT29, SW480, LOVO, and SW620) and normal colon epithelial cell line (NCM460) were obtained from KeyGEN BioTECH Corp (NanJing, China). Among them, HT29 was cultured in McCoy’s 5 A medium containing 10% fetal bovine serum (FBS); SW480 and SW620 were cultured in Leibovitz’s L15 medium containing 10% FBS; LOVO was cultured in Ham’s F-12K medium containing 10% FBS; and NCM460 was cultured in RPMI-1640 medium containing 10% FBS. All media and FBS were purchased from KeyGEN BioTECH Corp (Nanjing, China). All the cells were cultured at 37°C and 5% CO_2_ condition.

### qRT-PCR

Total RNA was isolated from the cells using TRIzol reagent (KeyGEN, NanJing, China), followed by cDNA synthesis using the cDNA synthesis kit (TaKaRa, Japan). Then, qRT-PCR was performed using 2× Real-time PCR Master Mix (SYBR Green) with GAPDH as an internal control. The primers used for PLA1A were as follows: forward: 5’-TCTGGGTTCAATGCCACTCT-3’ and reverse: 3’-CCACGGCAATCACATTAGCA-5’. The relative expression level of PLA1A was calculated using the 2^^-ΔΔCt^ method.

### Cell proliferation, invasion and apoptosis assays

To explore the function of PLA1A *in vitro*, overexpression vector (OE-PLA1A) was constructed using the pcDNA3.1(+) plasmid. The siRNA sequence targeting PLA1A (siPLA1A) used in this study was as follows: sense: 5’-GGAUAGGACUGGUGGAACATT-3’ and antisense: 5’-UGUUCCACCAGUCCUAUCCTT-3’. Using Lipofectamine 3000 reagent (Invitrogen, USA), the OE-PLA1A vector was transfected into HT29 cell line, while the siPLA1A was transfected into SW620 cell line. The corresponding negative control plasmid or siRNA was transfected into each cell line.

To detect the functional changes of CRC cells upon PLA1A overexpression or knockdown, cell proliferation, invasion and apoptosis assays were performed. For the cell proliferation assay, the cells transfected for 24 hours were added to a 96-well cell culture plate. Cell viability was measured at three incubation time points (24 h, 48 h, 72 h) using the Cell Counting Kit-8 (CCK-8, KeyGEN Biotechnology, China). The optical density (OD) values at 450 nm were then measured using a microplate reader (TECAN SPARK, Switzerland). For the cell invasion assay, serum was removed from cells transfected for 72 hours, and the cells were starved in serum-free medium for 2 hours. Next, 30 μL of diluted Matrigel was added to the upper chamber of the Transwell (Corning, USA), and 100 µL of cell suspension was placed in the Transwell chamber, with 500 µL of medium containing 20% FBS added to the lower chamber. After 24 hours of incubation, the invaded cells were then stained with 500 µL of 1× crystal violet in a 24-well plate. Finally, the number of invasive cells was counted by randomly selecting three fields of view under a microscope. For the cell apoptosis assay, the cells transfected for 72 hours were stained with Annexin-V APC and 7-AAD according to the manufacturer’s instructions (KeyGEN Biotechnology, China). The apoptosis rates of SW620 and HT29 cells were then detected by flow cytometry.

All experiments were independently performed at least three times, and the results are presented as the mean ± standard deviation. Statistical analysis and data visualization were conducted using GraphPad Prism 9.5. A non-paired Student’s t-test was used to compare the differences between two groups, with *P* < 0.05 considered statistically significant.

## Results

### Identification of ICD-related genes in CRC based on single-cell and bulk RNA-Seq data

**Figure 2:**
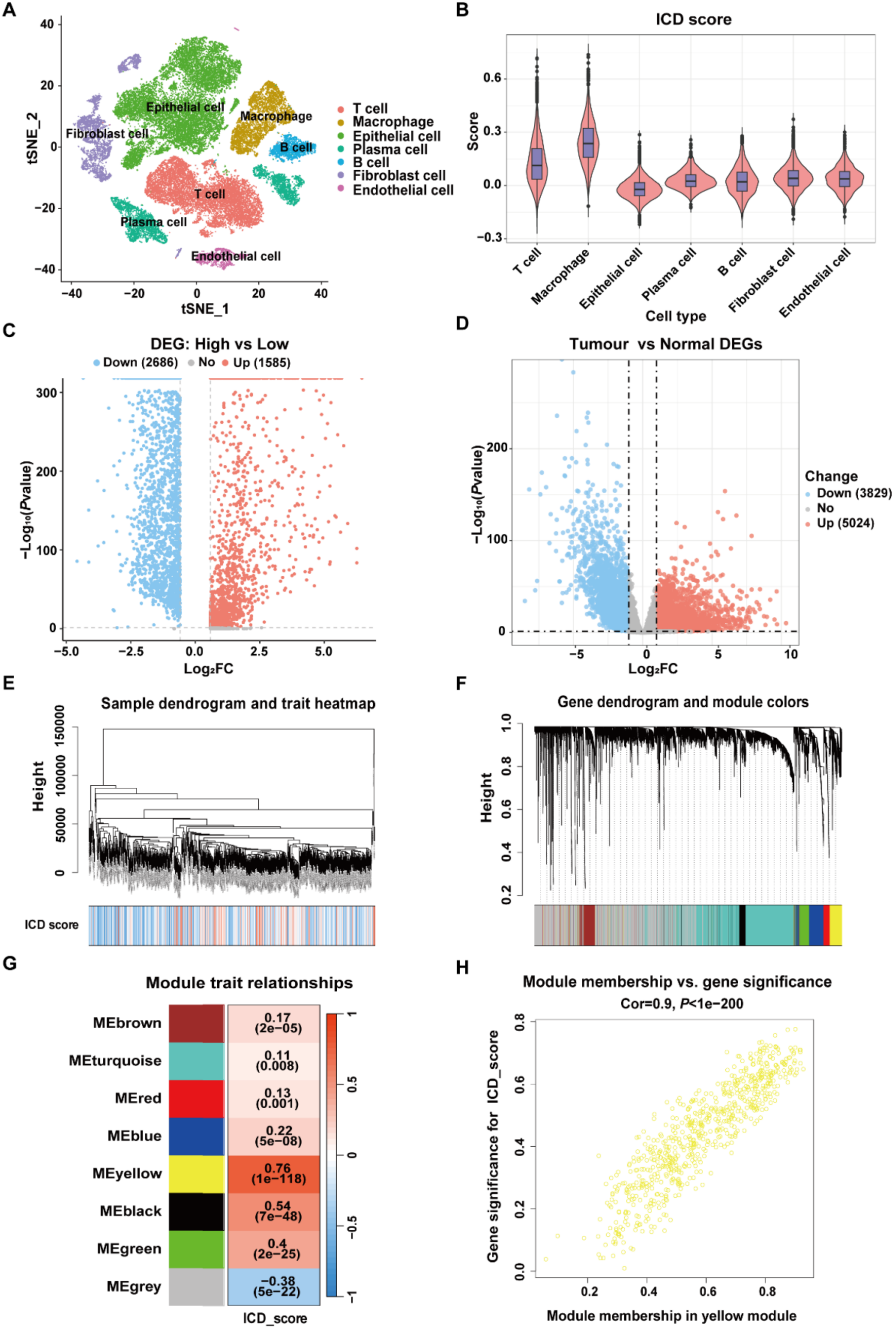
Identification of ICD-related genes in CRC. **(A)** t-SNE plot showing the distribution of cell types annotated based on common cell-specific marker genes. **(B)** Comparison of the ICD scores across different cell types. **(C)** Volcano plot showing the DEGs between high-ICD and low-ICD groups in GSE132465 dataset. **(D)** Volcano plot showing the DEGs between normal and tumor samples in TCGA-CRC dataset. **(E)** Dendrogram showing the hierarchical clustering of TCGA-CRC samples, with the ICD score for each sample represented in the heatmap at the bottom. **(F)** Cluster dendrogram from the WGCNA analysis performed on the TCGA-CRC dataset. **(G)** Module-trait heatmap showing the correlation between each module and the ICD score. **(H)** Scatter plot showing the relationship between gene significance (GS) and module membership (MM) in the yellow module.

To achieve a comprehensive molecular landscape between ICD and different cell populations in TME, scRNA-Seq data consisting of 41,958 cells from primary CRC (n=23) and matched normal mucosa (n=10) were obtained from GSE132465 dataset. After quality control and removing batch effects, PCA and t-SNE were applied on the top 1000 highly variable genes for dimensionality reduction. The single cells were clustered into 34 distinct clusters with a resolution of 0.5. As shown in **Figure 2A**, the cells were annotated into seven groups: T cells, macrophages, epithelial cells, plasma cells, fibroblast cell and endothelial cell, based on corresponding canonical marker genes. The expression patterns of three representative markers of each cell type, as well as the top three genes with the highest expression levels within each cell type were presented in **Supplementary Figure S1A** and **Supplementary Figure S1B**. Besides, the proportions of macrophages and epithelial cells were notably increased in CRC samples compared with normal tissue samples (**Supplementary Figure S1C**).

The expression levels of 34 ICD-related genes were calculated using the “AddModuleScore” function in the Seurat package to quantify the activity of ICD in the seven cell clusters. As shown in **Figure 2B**, macrophages exhibited the highest ICD activity among the seven cell types, followed by T cells. All cells were subsequently categorized into high-ICD and low-ICD groups based on the median ICD activity. Furthermore, 4,897 DEGs were identified between the high- and low-ICD groups with the cutoff of |log_2_FC|>0.585 and *P*.adj<0.05 (**Figure 2C**).

Next, differential expression analysis was performed using bulk RNA-Seq data to explore pivotal genes involved in CRC development. As shown in **Figure 2D**, 8,853 DEGs were identified between tumor and normal tissues in the TCGA-CRC dataset with the cutoff of |log_2_FC|>0.585 and *P*.adj<0.05. Moreover, gene modules associated with ICD in the TCGA-CRC dataset were identified through WGCNA, with the phenotype data being the ICD score calculated using the ssGSEA algorithm. (**Figure 2E**). Among the eight identified modules, the MEyellow module, comprising 682 genes, showed the highest correlation with the ICD score (cor=0.76, *P*=1e-118) (**Figures 2F, G**). Additionally, there was a significant correlation between the gene significance (GS) and module membership (MM) for the MEyellow module (cor=0.9, *P*=1e-200) (**Figure 2H**), suggesting that the genes within the MEyellow module were potentially associated with ICD.

**Figure 3:**
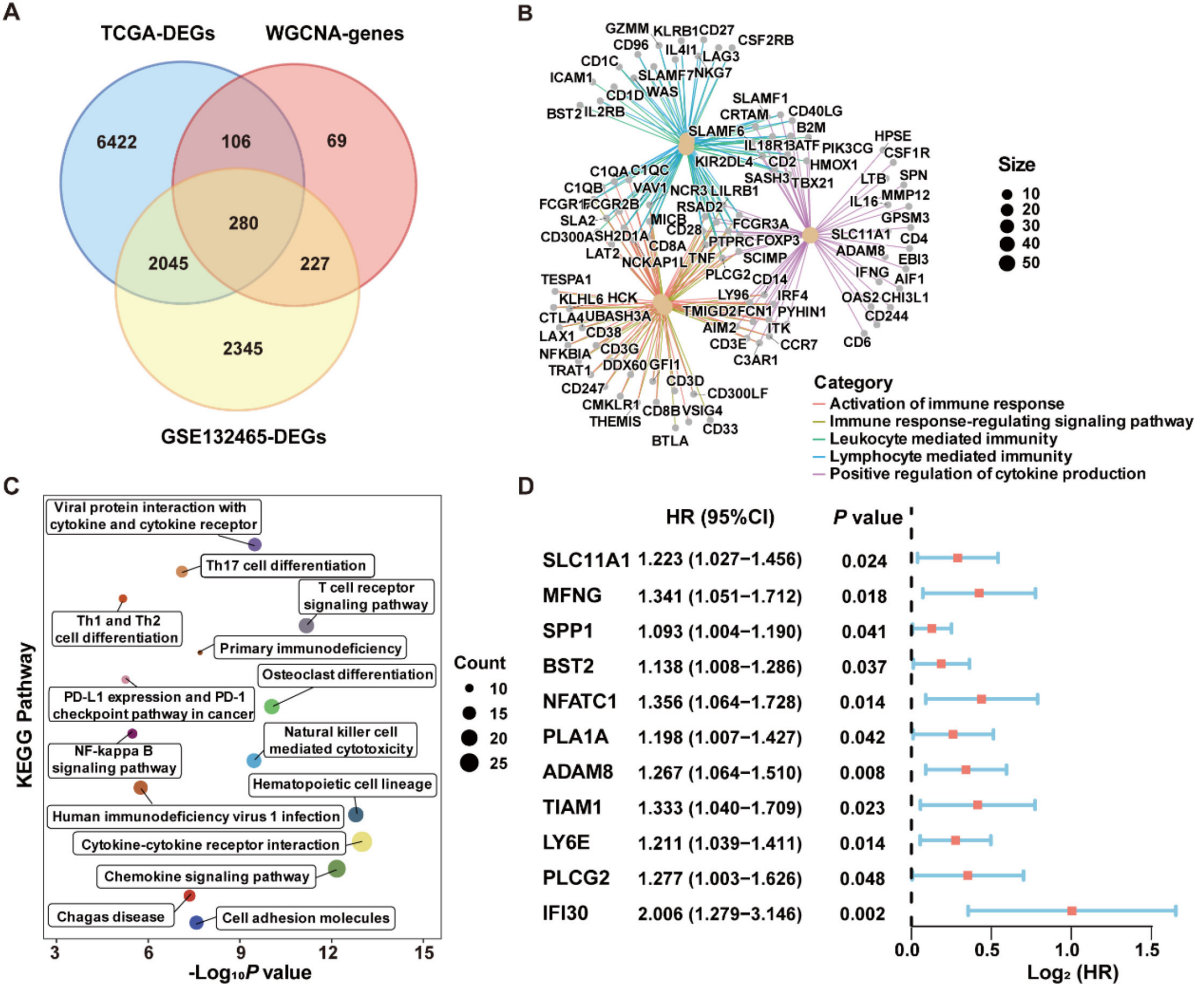
Identification of ICD-related genes associated with prognosis in CRC. **(A)** Venn diagram showing the intersection of genes among DEGs from the scRNA-Seq dataset GSE132465, DEGs from bulk RNA-Seq dataset TCGA-CRC, and the genes within the MEyellow module identifed through WGCNA analysis. **(B)** GO enrichment analysis of the ICD-related genes. **(C)** KEGG enrichment analysis of the ICD-related genes. **(D)** Univariate Cox regression analysis of ICD-related genes with prognostic value.

Finally, a total of 280 ICD-related genes in CRC were overlapped among the DEGs between high- and low-ICD groups in the GSE132465, the DEGs between tumor and normal tissues in the TCGA-CRC dataset, and the genes within the ICD-related MEyellow module (**Figure 3A**). GO enrichment analysis indicated that these ICD-related genes were involved in immune-related biological process terms, including the activation of immune response, regulation of immune response signaling pathway, immune processes mediated by leukocytes and lymphocytes, as well as positive regulation of cytokine production (**Figure 3B**). KEGG analysis further indicated that these genes participate in pathways such as T cell receptor signaling, the NF-κB signaling, and the PD-L1 expression and PD-1 checkpoint pathway in cancer (**Figure 3C**).

### Robust ICDRS modelling based on an integrated machine learning framework

**Figure 4:**
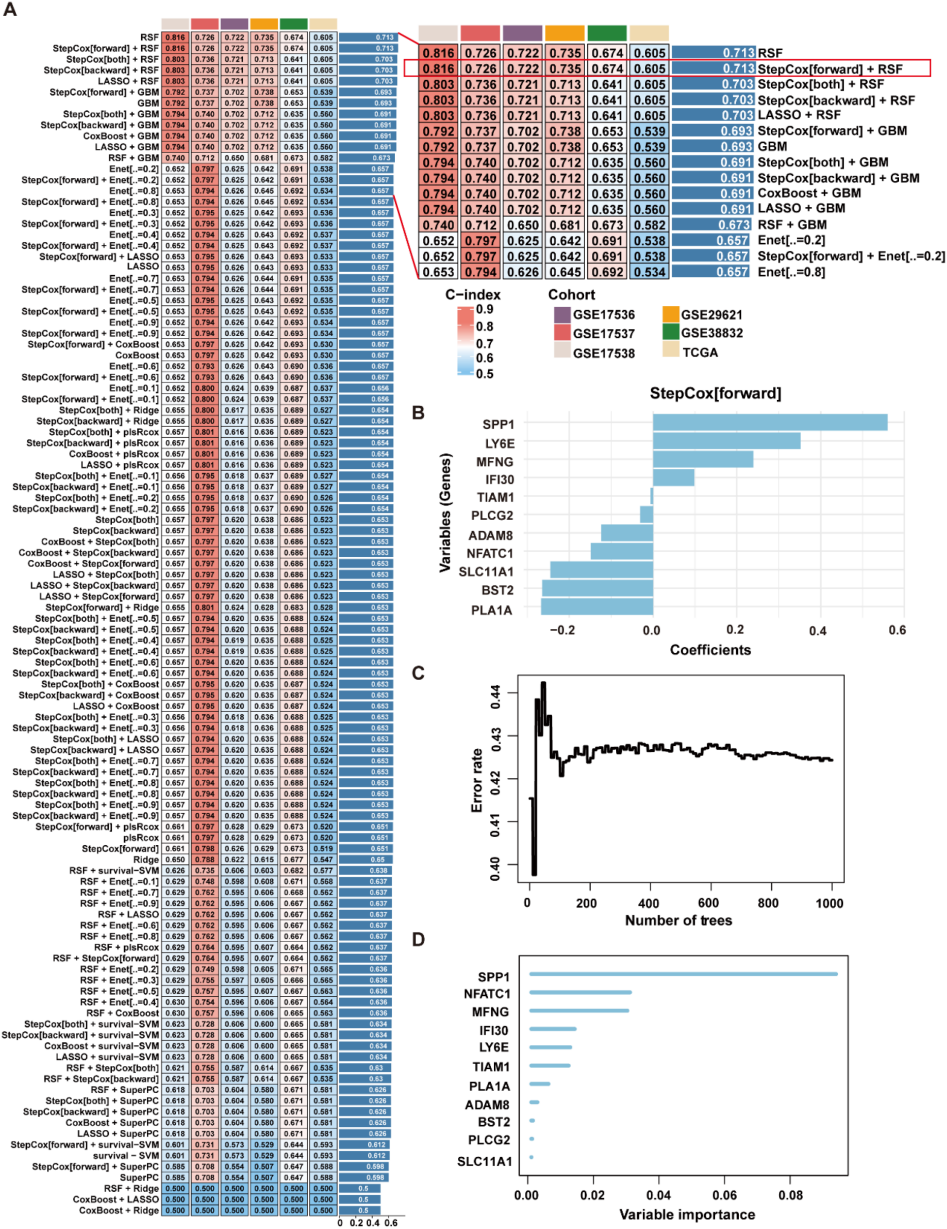
Construction of the ICDRS through a comprehensive machine learning framework containing 101 algorithm combinations. **(A)** The C-index value of each combinatorial algorithm on the training and validation sets was calculated, with the StepCox[forward]+RSF algorithm ultimately selected as the optimal method. **(B)** The coefficients for the 11 genes comprising ICDRS calculated by the StepCox[forward] algorithm. **(C)** The out-of-bag error of the RSF algorithm. **(D)** The importance of the 11 genes comprising the ICDRS, as calculated by RSF algorithm.

To construct a prognostic signature for CRC, univariate Cox analysis was conducted to identify prognostic genes among the 280 ICD-related genes. As shown in **Figure 3D**, 11 ICD-related genes were identified with significant prognostic value in CRC (*P*<0.05). The 11 ICD-related genes were then used as input to develop prognostic models using machine learning algorithms with 101 combinations, and the model constructed by StepCox[forward] + RSF combination achieved the highest average C-index of 0.713 across the training and validation datasets, and is therefore considered to be the optimal approach for developing ICDRS (**Figure 4A**). The high C-index of the StepCox[forward] + RSF model highlights its robust predictive performance in assessing prognostic risk in CRC patients, outperforming all other evaluated algorithmic combinations. In detail, ICDRS comprises 11 genes, including SLC11A1, MFNG, SPP1, BST2, NFATC1, PLA1A, ADAM8, TIAM1, LY6E, PLCG2, and IFI30. The coefficients and relative importance of these genes were determined through StepCox[forward] and RSF, respectively (**Figures 4B–D**). First, variable selection was performed using the “StepCox[forward]” algorithm, followed by the extraction of important variables which were then incorporated into the “RSF” algorithm to calculate risk scores. Finally, all patients were divided into high- and low-risk groups according to the median of the risk scores.

### Evaluation of the predictive performance of ICDRS

**Figure 5:**
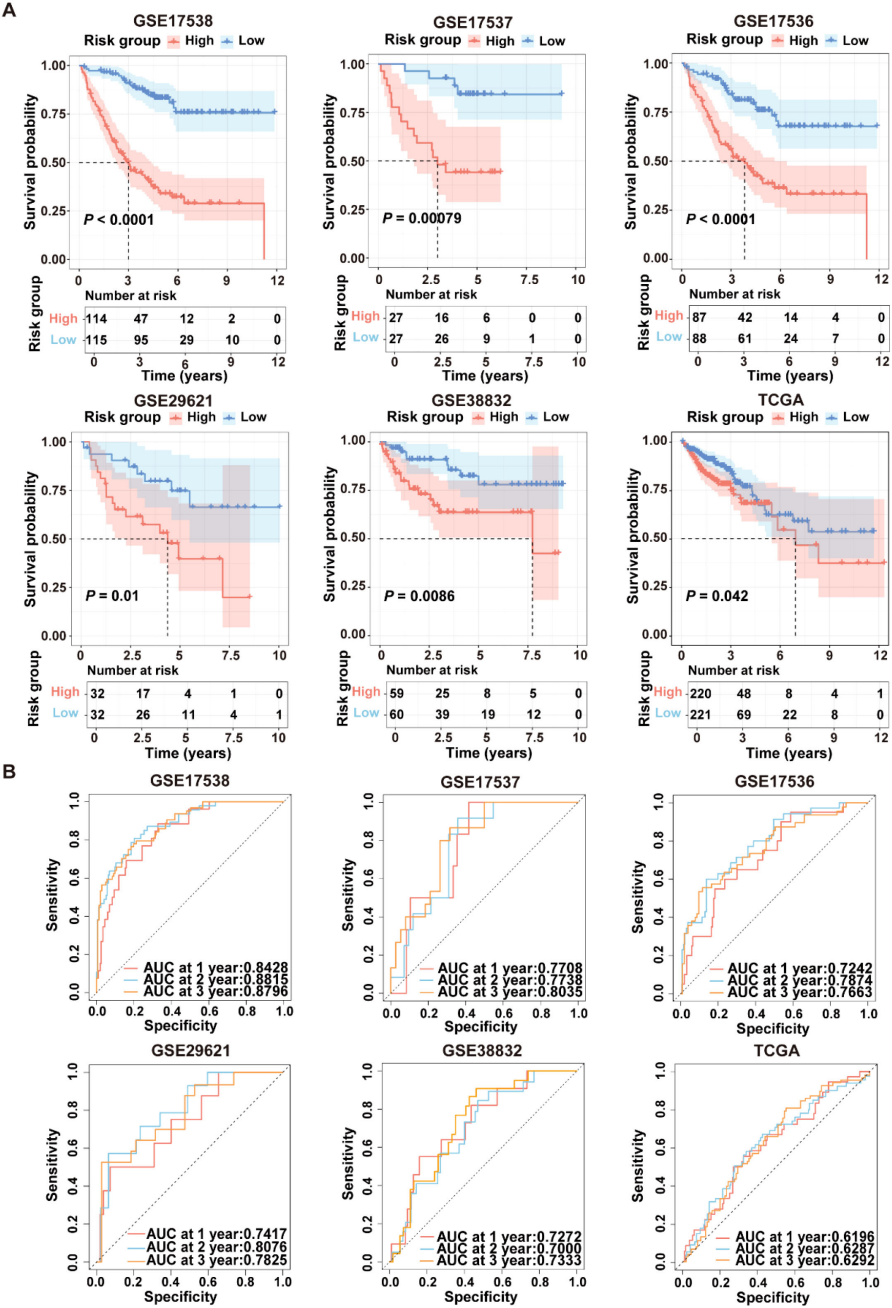
Performance evaluation of the ICDRS in both the training and validation datasets. **(A)** Kaplan-Meier curve analyses were performed to evaluate the performance of the ICDRS in predicting OS in the GSE17538, GSE17537, GSE17536, GSE29621, and TCGA-CRC datasets, as well as DFS in the GSE38832 dataset. **(B)** ROC curve analyses were conducted to assess the specificity and sensitivity of ICDRS for predicting 1-, 2- and 3-year OS in the GSE17538, GSE17537, GSE17536, GSE29621, and TCGA-CRC datasets, and for predicting 1-, 2- and 3-year DFS in the GSE38832 dataset.

To evaluate the robustness of ICDRS performance in predicting survival outcomes, CRC patients in the training and validation datasets were stratified into high- and low-risk subgroups based on the median risk score. As shown in **Figure 5A**, Kaplan-Meier curve analysis demonstrated that patients with high-risk had significantly lower OS rates in GSE17538, GSE17537, GSE17536, GSE29621, GSE38832, and TCGA-CRC compared to patients with low-risk (all *P*<0.05). Moreover, time-dependent ROC curve analysis demonstrated that the ICDRS exhibited good predictive performance for OS in CRC patients, with area under the curve (AUC) values ranging from 0.8428 to 0.8796 at 1, 2, and 3 years (**Figure 5B**). Subsequently, multivariate Cox regression analysis was performed in the training and validation datasets, taking ICDRS-derived risk score along with common clinical factors as variables. The results showed that the risk score could serve as an independent prognostic factor for OS but not for DFS in CRC (**Supplementary Figure S2**). Furthermore, to comprehensively compare the performance of the ICDRS with other prognostic signatures in CRC, 60 published signatures were collected (**Supplementary Figure S3**). Notably, ICDRS achieved the highest C-index in GSE17538, GSE17537, GSE17536, GSE29621, and GSE38832 datasets. These findings highlight the robust predictive performance of the ICDRS.

### Development of a nomogram based on ICDRS and clinical factors for CRC

**Figure 6:**
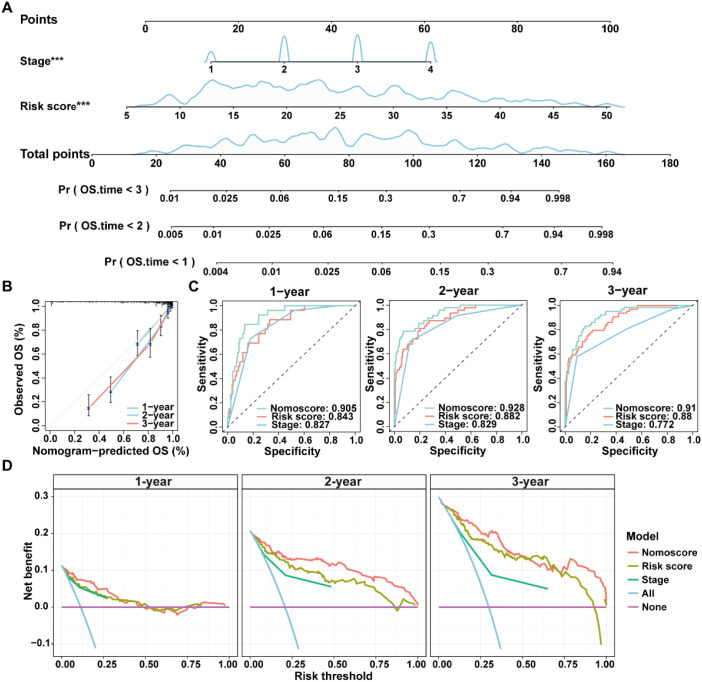
Construction and evaluation of a nomogram for CRC patients in the GSE17538 dataset. **(A)** A nomogram was constructed based on the ICDRS-derived risk score and the disease stage. **(B)** Calibration curves for the 1-, 2-, and 3-year OS predictions from the nomogram. **(C)** ROC curves showing the performance of the nomogram in predicting the 1-year, 2-year, and 3-year OS. **(D)** DCA curves showing the clinical benefit of the nomoscore, risk score and stage to predict the prognosis of CRC patients at 1-, 2-, and 3-year. Nomoscore, nomogram-derived score. ***Indicates that the P value is less than 0.001 in the multivariate Cox regression.

Given the advantage of nomogram in simplify risk prediction models into numerical estimates of mortality, a nomogram was constructed by incorporating age, gender, clinical stage, and ICDRS-based risk score to assess the 1-, 2-, and 3-year survival probability for CRC patients (**Figure 6A**). The calibration curves demonstrated a high consistency between the predicted values of the nomogram and the actual observed outcomes (**Figure 6B**). Moreover, the nomogram achieved AUCs of 0.905, 0.928, and 0.910 for predicting 1-year, 2-year, and 3-year survival, respectively, indicating its high predictive accuracy (**Figure 6C**). Decision curve analysis also revealed that the nomogram had a higher predictive benefit for clinical outcomes of CRC patients compared with other clinical features (**Figure 6D**). These results suggest that the ICDRS-based nomogram could serve as a reliable and accurate tool for personalized prognostic prediction in CRC patients.

### Close relationship between ICDRS and immune cell infiltration within the TME

**Figure 7:**
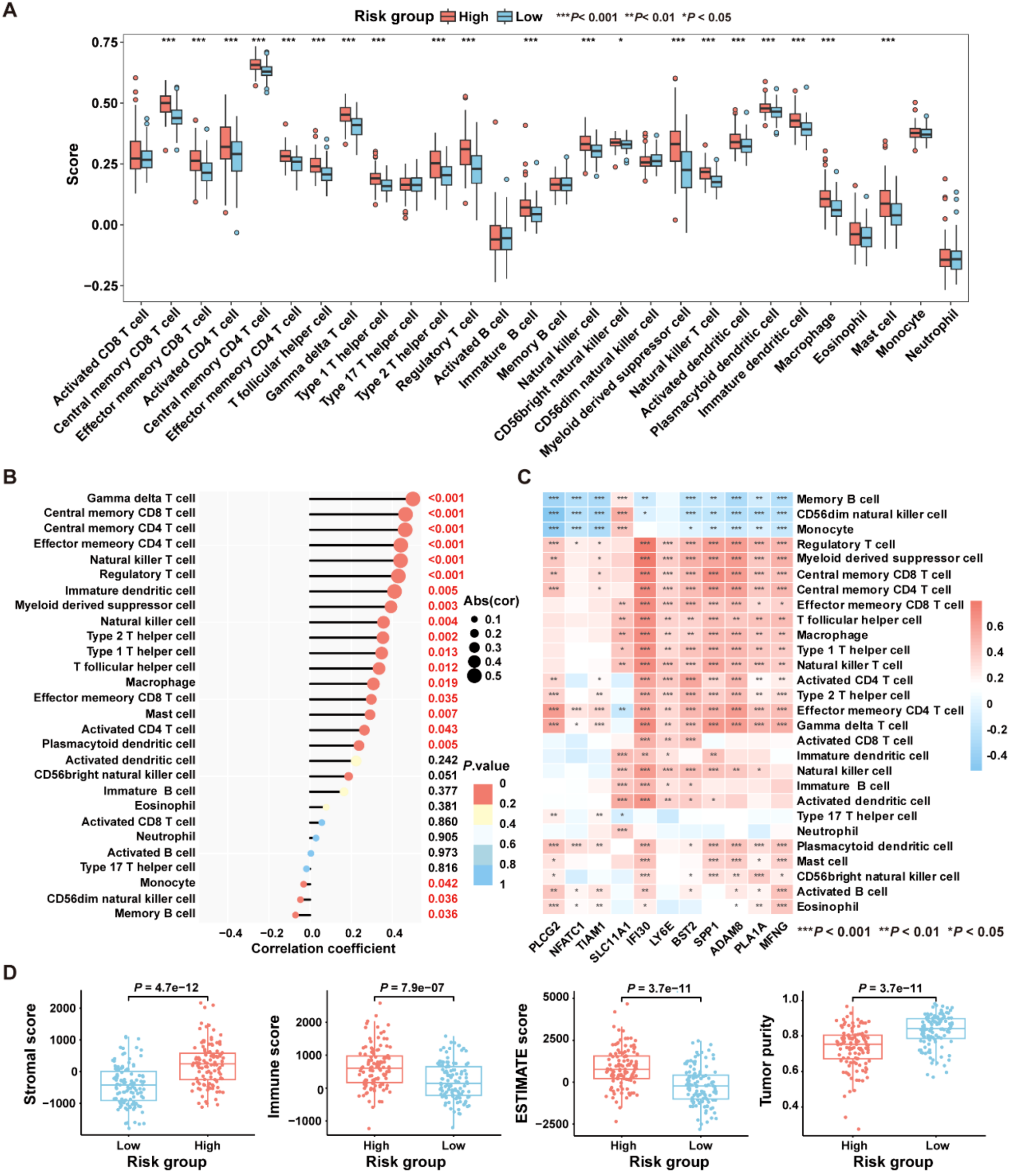
Comparative analysis of the immune characteristics between high-risk and low-risk groups in the GSE17538 dataset. **(A)** Box plots showing the immune cell scores in high- and low-risk groups as assessed by the ssGSEA algorithm. **(B)** The correlation between immune cell infiltration levels and risk score was analyzed using Pearson correlation coefficient. **(C)** The Pearson correlation coefficient was used to analyze the correlation between immune cell infiltration levels and the expression levels of the 11 genes comprising the ICDRS. **(D)** Comparison of immune states between high- and low-risk groups based on stromal score, immune score, ESTIMATE score, and tumor purity. ^*^ *P*<0.05, ^**^ *P*<0.01, ^***^ *P*<0.001.

To explore the relationship between the ICDRS and immune components within the TME, the ssGSEA algorithm was first utilized to quantify the abundance of infiltrating immune cells in each sample of the GSE17538 dataset, enabling an assessment of immune infiltration status across high- and low- risk groups. Results showed that the high-risk group exhibited a significantly higher enrichment of macrophages, regulatory T cells, natural killer T cells, and myeloid-derived suppressor cells compared to the low-risk group (**Figure 7A**). Moreover, a positive correlation was observed between the risk score and the abundance of most tumor-infiltrating immune cells, such as macrophage, regulatory T cells, natural killer T cells, immune dendritic cells, natural killer cells and myeloid-derived suppressor cells (**Figure 7B**). Besides, the infiltration levels of macrophages and natural killer T cells were positively correlated with the expression levels of SLC11A1, IFI30, LY6E, BST2, SPP1, ADAM8, PLA1A, and MFNG, respectively (*P*<0.05). In contrast, the infiltration level of memory B cells was negatively correlated with the expression levels of PLCG2, NFATC1, TIAM1, IFI30, BST2, SPP1, ADAM8, PLA1A, and MFNG, respectively (*P*<0.05) (**Figure 7C**).

Subsequently, the “ESTIMATE” algorithm was applied to calculate the immune score, stromal score, ESTIMATE score, and tumor purity score for the high-risk and low-risk groups within the GSE17538 dataset. Results showed that the high-risk group had significantly higher stromal score (*P*=4.7e-12), immune score (*P*=7.9e-07), and ESTIMATE score (*P*=3.7e-11) compared to the low-risk group, while the tumor purity score (*P*=3.7e-11) was significantly lower (**Figure 7D**). High immune scores coexisting with moderate or high stromal scores may be detrimental to the prognosis of CRC patients (32). These findings suggest that patients in the high-risk group are likely to have a worse prognosis than those in the low-risk group.

### ICDRS as a candidate predictor for immunotherapy response of CRC

**Figure 8:**
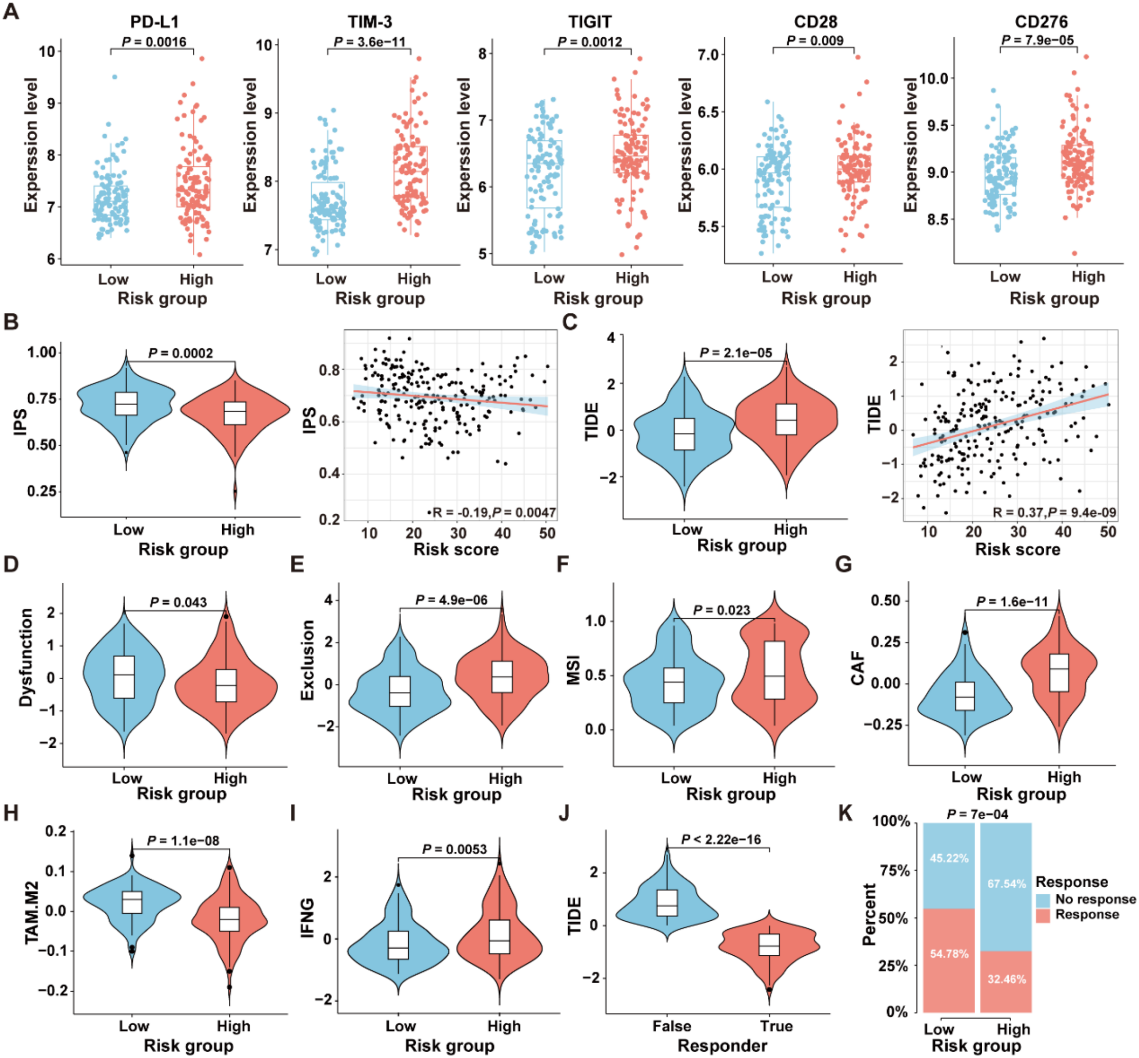
Comparison of the response to immunotherapy between high- and low-risk groups in the GSE17538 dataset. **(A)** Expression levels of immune checkpoint genes in the high- and low-risk groups. **(B)** The comparison of IPS scores between the high- and low-risk groups, along with the correlation between IPS scores and ICDRS-derived risk scores, was analyzed. **(C)** The comparison of TIDE scores between the high- and low-risk groups, along with the correlation between TIDE scores and ICDRS-derived risk scores, was analyzed. **(D–I)** The immune dysfunction score **(D)**, immune exclusion score **(E)**, microsatellite instability **(F)**, CAF level **(G)**, TAM-M2 level **(H)** and IFNG expression level **(I)** were compared between the high- and low-risk groups, respectively. **(J)** The comparison of TIDE scores between responders and non-responders to anti-PD1 and anti-CTLA4 immunotherapies. **(K)** Comparison of the proportions of the responsive and non-responsive patients who received immunotherapy in the high- and low-risk groups of the GSE17538 cohort.

To select patient populations that may benefit from immune checkpoint inhibitor (ICI) treatment, we first analyzed the expression levels of ICI-related genes in the high- and low-risk groups in the GSE17538 dataset. The results revealed significant differences in the expression levels of PD-L1 (*P*=0.0016), TIM-3 (*P*=3.6e-11), TIGIT (*P*=0.0012), CD28 (*P*=0.009), and CD276 (*P*=7.9e-05) between the two risk groups (**Figure 8A**). The IPS is a robust predictor of antibody responses targeting cytotoxic T lymphocyte antigen-4 (CTLA-4) and programmed cell death protein 1 (PD-1) (21). To further analyze the differences in immunotherapy responses between high-risk and low-risk groups, the “IOBR” R package was employed to predict the IPS of patients in the GSE17538 dataset. The results revealed a significant difference in the IPS between the high-risk and low-risk patient groups (*P*=0.0002), with a negative correlation observed between the IPS and the risk score (R=-0.19, *P*=0.0047) (**Figure 8B**), suggesting that patients in the low-risk group might obtain greater benefits from immunotherapeutic interventions.

Moreover, the TIDE analysis was conducted to evaluate the differences between the high-risk and low-risk groups in terms of tumor immune evasion mechanisms. As shown in **Figure 8C**, the TIDE scores of the high-risk group are significantly higher than those of the low-risk group (*P*=2.1e-05), and a positive correlation was observed between the TIDE scores and the risk scores (R=0.37, *P*=9.4e-09). Since higher TIDE scores are associated with a poorer response to immune checkpoint inhibition therapy, these results suggest that patients in the low-risk group may be more suitable candidates for immunotherapy. Additionally, there were significant differences in the TME-related scores, including dysfunction score (*P*=0.0043), exclusion score (*P*=4.9e-06), MSI score (*P*=0.023), cancer-associated fibroblast (CAF) score (*P*=1.6e-1), TAM.M2 score (*P*=1.1e-08), and IFNG score (*P*=0.0053) between the high-risk and low-risk groups in GSE17538 (**Figures 8D–I**). In addition, patients were classified into responsive and non-responsive groups based on their clinical response to anti-PD1 and anti-CTLA4 immunotherapies (33). As shown in **Figure 8J**, the TIDE scores in the non-responsive group of the GSE17538 dataset were significantly higher than those in the responsive group (*P*<2.22e-16), and the proportion of responders in the low-risk group was significantly higher than that in the high-risk group (**Figure 8K**, *P*=7e-04). These findings collectively indicate that high-risk CRC patients tended to encounter immune evasion when receiving immunotherapy than those in the low-risk group.

### Genomic variation landscape across ICDRS-defined subgroups

**Figure 9:**
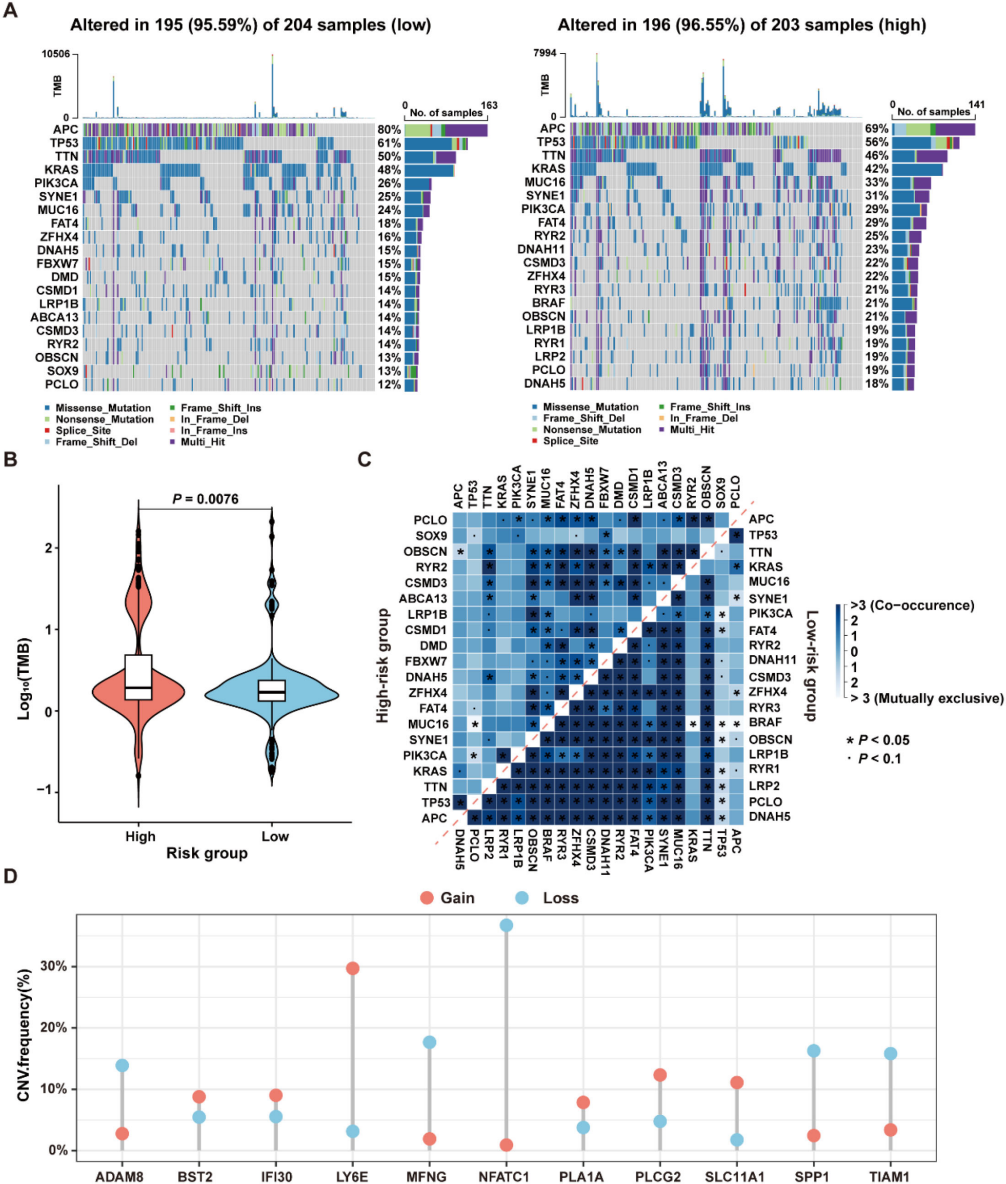
Genomic variation landscape in low- and high-risk groups in the TCGA-CRC dataset. **(A)** Waterfall plots of somatic mutation landscapes in the low-risk group (left) and high-risk group (right). **(B)** Violin plots showing the distribution of TMB values in the high-risk and low-risk groups. **(C)** The top 20 genes exhibiting either co-occurrence or mutual exclusivity in mutation patterns, were observed in the high-risk and low-risk groups, respectively. **(D)** The distribution of CNV frequencies for the 11 genes that constitute the ICDRS.

To investigate the differences in genomic mutations between high-risk and low-risk groups in the TCGA-CRC dataset, the TMB analyses were conducted for each group separately. The results revealed that the top twenty genes with the highest mutation frequencies were entirely different between the two groups, with the high-risk group exhibiting a higher mutation frequency than the low-risk group (**Figure 9A**). Moreover, since CRC patients with high TMB may benefit from ICI therapy (34), the TMB values were compared between the high- and low-risk groups. As shown in **Figure 9B**, TMB values in the high-risk group were significantly higher than those in the low-risk group (*P*=0.0076), suggesting that patients in the high-risk group may be more suitable for ICI treatment.

The reasons for the different prognostic states among subgroups were explained by analyzing the correlation between co-occurring and mutually exclusive mutations in the top 20 mutated genes within the high-risk and low-risk groups. As shown in **Figure 9C**, there were significant differences in the number and types of co-occurring mutation gene pairs between the high-risk and the low-risk groups, suggesting that these genes may be key factors involved in regulating the distinct mutational landscapes of the two groups. Additionally, the presence of CNV in the 11 genes comprising the ICDRS was examined. The results showed that ADAM8, BST2, IFI30, LY6E, MFNG, PLA1A, PLCG2, SLC11A1, SPP1, and TIAM1 exhibited both deletions and amplifications, while NFATC1 had a significant copy number deletion (**Figure 9D**). These findings suggest that CNV alterations play a potential role in regulating gene expression.

### Functional analysis of the key signature gene PLA1A in the ICDRS

**Figure 10:**
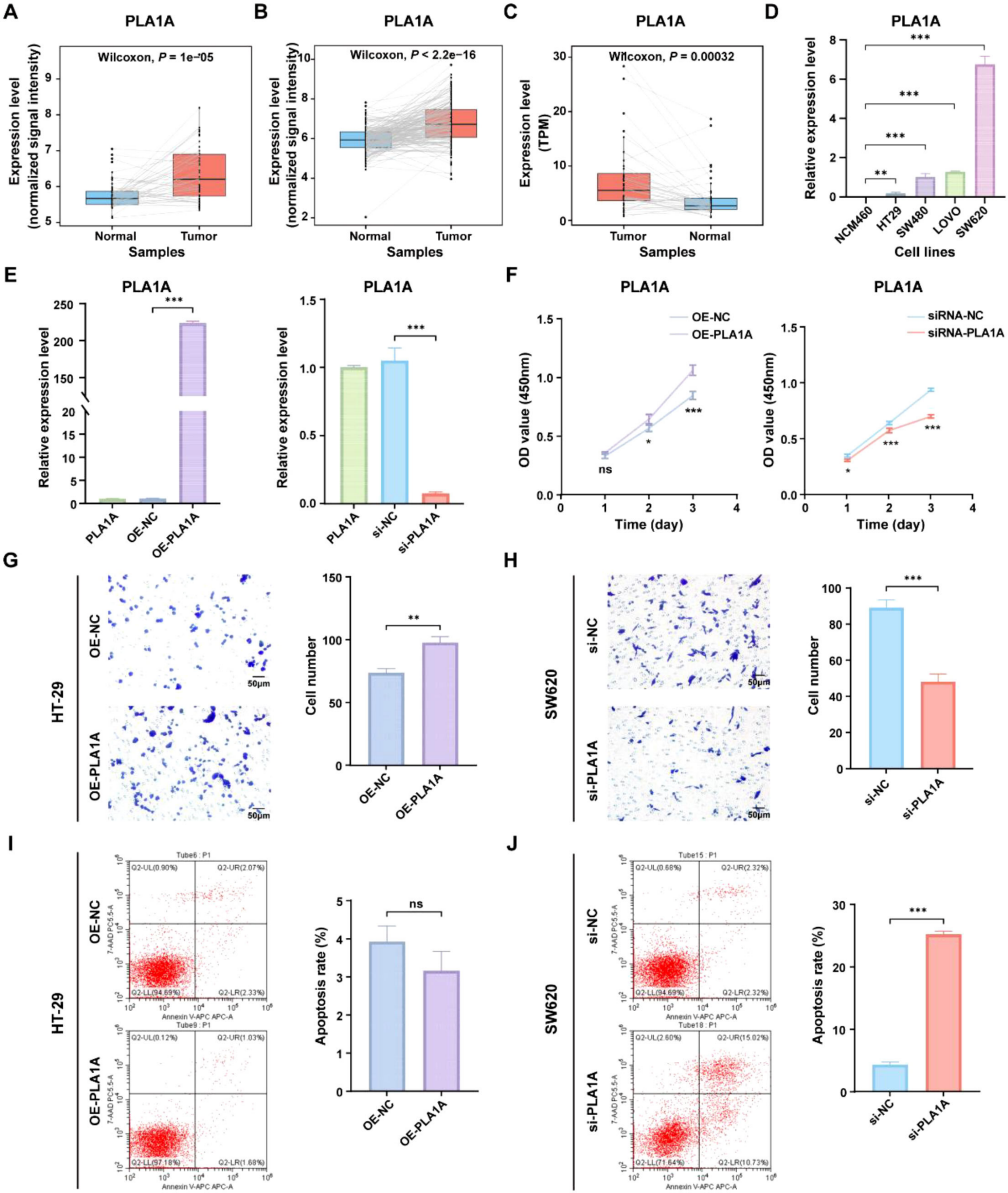
Functional analysis of the PLA1A gene in ICDRS. **(A–C)** Comparison of the PLA1A expression level between paired tumor and normal control samples in the GSE44861 **(A)**, GSE87211 **(B)** and TCGA-CRC **(C)** datasets. **(D)** The expression level of PLA1A was detected by qRT-PCR in human normal colon epithelial cell line NCM460 and CRC cell lines (HT-29, SW480, LOVO, and SW620). **(E)** The efficiency of PLA1A overexpression in HT-29 cells and the of siRNA-mediated PLA1A interference in SW620 cells were detected by qRT-PCR. **(F)** The effects of PLA1A overexpression on HT29 cell proliferation and its knockdown on SW620 cell proliferation were evaluated using the CCK-8 assay. **(G, H)** The effects of PLA1A overexpression on HT29 cell invasion and its knockdown on SW620 cell invasion were assessed using the Transwell assay. **(I, J)** The effects of PLA1A overexpression on apoptosis in HT29 cells and its knockdown on apoptosis in SW620 cells were assessed using apoptosis assays. ^*^ *P*<0.05, ^**^ *P*<0.01, ^***^ *P*<0.001.

To gain deeper insights into the role of ICDRS in CRC, we aimed to perform a functional analysis of the genes that constitute the ICDRS. A literature review revealed that the function of PLA1A within this gene set has not been reported in CRC, highlighting its novelty. Additionally, PLA1A showed significant upregulation in tumor tissues compared with paracancerous tissues in three independent CRC cohorts including GSE44861, GSE87211, and TCGA-CRC (**Figures 10A–C**), suggesting its potential biological relevance in CRC progression. This upregulation was further confirmed by qRT-PCR analysis, which demonstrated significantly higher PLA1A expression in CRC cell lines (HT-29, SW480, LOVO, and SW620) compared to normal human colon mucosal epithelial cell line (NCM460), with the highest expression observed in the SW620 cell line (*P* < 0.05, **Figure 10D**). Therefore, the functional role of PLA1A was further investigated in this study.

To investigate the impact of PLA1A expression changes on CRC cells, PLA1A was sucessfully overexpressed in HT29 cell lines and knocked down in SW620 cell line (**Figure 10E**), which were then used for subsequent *in vitro* experiments. As shown in **Figures 10F–H**, cell viability and invasion ability of HT29 cells in the OE-PLA1A group were significantly higher than that in the OE-NC group. Similarly, cell viability and invasion ability of SW620 cells in the si-PLA1A group were significantly lower than that in the si-NC group. Besides, although the total apoptosis rate of HT-29 cells in the OE-PLA1A group remained largely unchanged (**Figure 10I**), the total apoptosis rate of SW620 cells in the siRNA-PLA1A group was significantly increased (**Figures 10J**, *P* < 0.001). These findings indicate that PLA1A may play a crucial role in CRC progression, helping us understand the translational significance of the ICDRS.

### Macrophage subgrouping based on ICD-related prognostic genes

**Figure 11:**
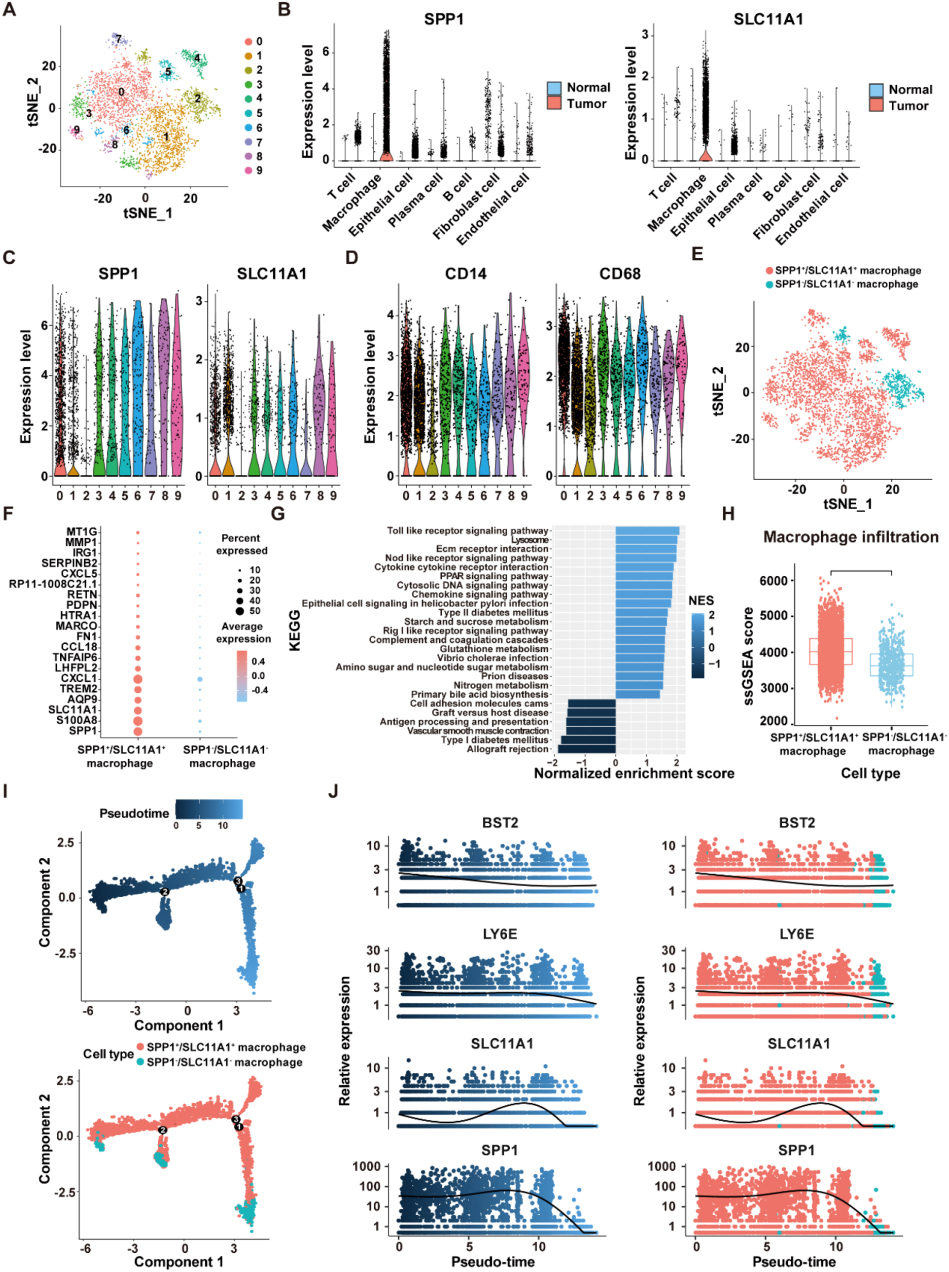
Identification of SPP1^+^/SCL11A1^+^ macrophages in the GSE132465 dataset. **(A)** t-SNE plot showing 10 macrophage sub-clusters identified at a resolution of 0.5. **(B)** Violin plots displaying the expression levels of SPP1 and SLC11A1 in different cells of tumor and normal tissues. **(C)** The expression levels of SPP1 and SLC11A1 in 10 macrophage sub-clusters. **(D)** The expression levels of CD14 and CD68 in 10 macrophage sub-clusters. **(E)** tSNE plot showing the distribution of SPP1^+^/SCL11A1^+^ and SPP1^-^/SCL11A1^-^ macrophages. **(F)** The top 20 DEGs between SPP1^+^/SCL11A1^+^ macrophages and SPP1^-^/SCL11A1^-^ macrophages. **(G)** GSEA analysis of DEGs between SPP1^+^/SCL11A1^+^ macrophages and SPP1^-^/SCL11A1^-^ macrophages. **(H)** Comparison of infiltration levels of SPP1^+^/SCL11A1^+^ and SPP1^-^/SCL11A1^-^ macrophages assessed by the ssGSEA algorithm. **(I)** The differentiation trajectory of SPP1^+^/SCL11A1^+^ and SPP1^-^/SCL11A1^-^ macrophages. **(J)** Dynamic changes of BST2, LY6E, SLC11A1 and SPP1 levels during macrophage differentiation.

As the highest activity of ICD genes was observed in macrophages, we focused on analyzing the role of ICDRS in these cells. Based on the expression levels of the 11 genes that make up the ICDRS, macrophages were divided into 10 subclusters in the scRNA-Seq dataset GSE132465 utilizing the “t-SNE” algorithm (**Figure 11A**). Notably, SPP1 and SLC11A1 were expressed almost exclusively in macrophages (**Figure 11B**), suggesting their potential as biomarkers for macrophage-related processes. Accordingly, based on the expression pattern of SPP1 and SLC11A1 in each cell cluster, subclusters 0–1 and 3–9 with high expression levels of both genes were classified as SPP1^+^/SLC11A1^+^ macrophages, while the subcluster 2 with no expression of both genes was classified as SPP1^-^/SLC11A1^-^ macrophages (**Figures 11C, E**). The expression patterns of SPP1 and SLC11A1 in the identified macrophage subtypes were different from classic macrophage markers CD14 and CD68 (**Figure 11D**).

To investigate the differences in biological functions between the two macrophage subtypes, DEGs analysis was performed using the “FindMarkers” function (**Figure 11F**). Subsequently, GSEA analysis revealed that the upregulated genes were significantly enriched in the Toll-Like receptor signaling pathway, NOD-Like receptor signaling pathway, and cytokine-cytokine receptor interaction. Comparatively, the downregulated genes were enriched in the allograft rejection, vascular smooth muscle contraction, and antigen processing and presentation pathways (**Figure 11G**). Moreover, macrophage infiltration level was significantly higher in the SPP1^+^/SLC11A1^+^ group compared to the SPP1^-^/SLC11A1^-^ group (*P*<2.22e-16) (**Figure 11H**). The transformation of macrophage fate likely originates from SPP1^+^/SLC11A1^+^ macrophages, which then develop into SPP1^-^/SLC11A1^-^ macrophages, as suggested by the pseudo-time sequence. During this differentiation process, dynamic changes were observed in the expression levels of BST2, LY6E, SLC11A1, and SPP1 (**Figures 11I, J**). Overall, these findings highlight that SPP1^+^/SLC11A1^+^ macrophages are functionally distinct from SPP1^-^/SLC11A1^-^ macrophages, with potential implications for immune-related processes.

**Figure 12:**
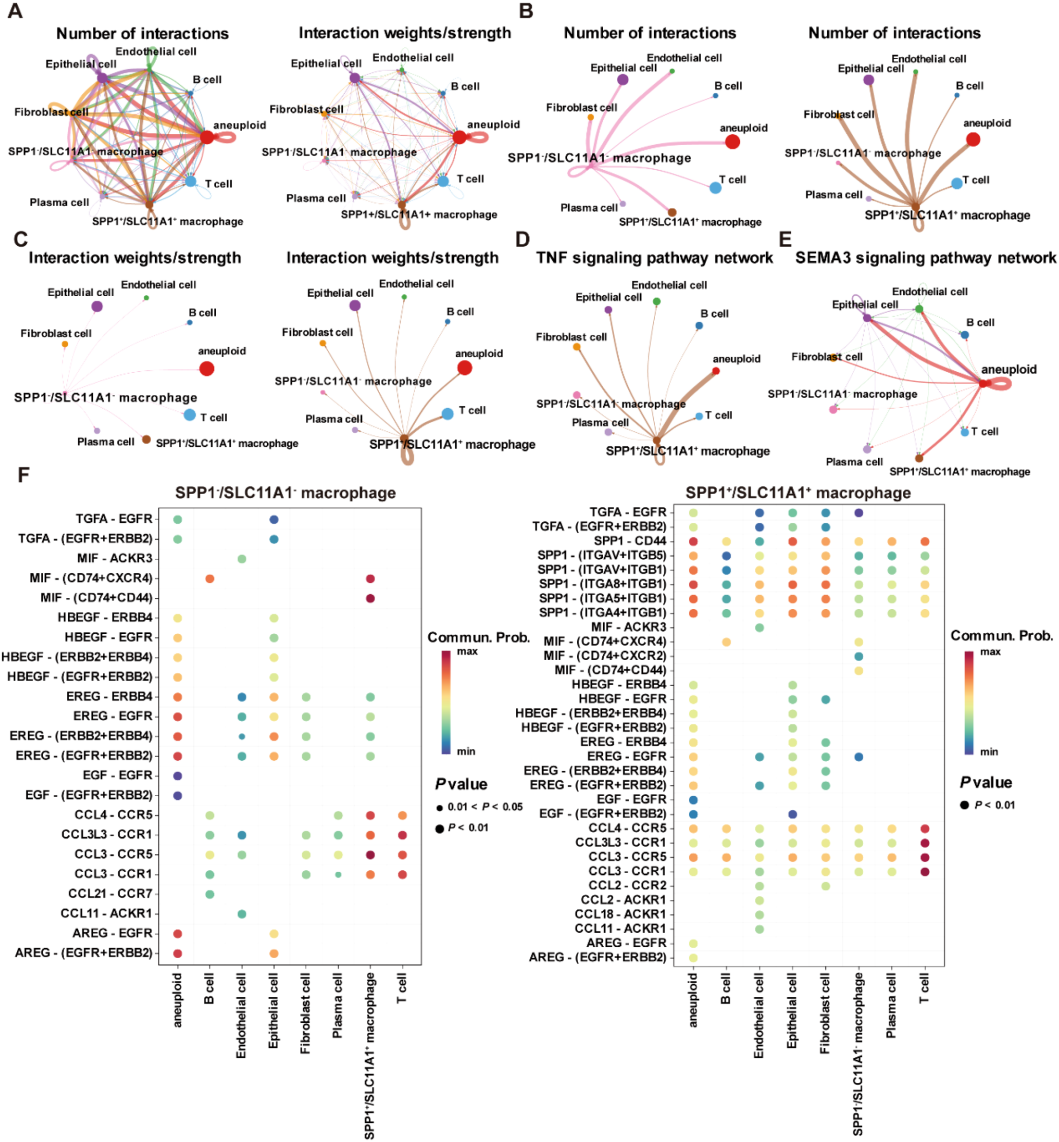
Cell-cell communication within the TME in the GSE132465 dataset. **(A)** The number of interactions and interaction weights/strength between cells in the TME. **(B)** The number of interactions between SPP1^-^/SCL11A1^-^ macrophages and other major cell types in the TME (left), and the number of interactions between SPP1^+^/SCL11A1^+^ macrophages and other major cell types in the TME (right). **(C)** The interaction weights/strength between SPP1^-^/SCL11A1^-^ macrophages and other major cell types in the TME (left), and the interaction weights/strength between SPP1^+^/SCL11A1^+^ macrophages and other major cell types in the TME (right). **(D)** The TNF signaling pathway network in the communication between SPP1^+^/SCL11A1^+^ macrophages and other cells in the TME. **(E)** The SEMA3 signaling pathway network in the communication between aneuploid cells and other cells in the TME. **(F)** Bubble plots showing the key outgoing and incoming signaling patterns of the SPP1^-^/SCL11A1^-^ macrophages (left) and SPP1^+^/SCL11A1^+^ macrophages (right).

Moreover, copy number analysis was performed to identify aneuploid cells in the GSE132465 dataset, showing that aneuploid cells were primarily located within the epithelial cell population, and they could contribute to the development and progression of CRC due to their genomic instability (**Supplementary Figures S4A, B**). Cell communication analysis revealed that SPP1^+^/SLC11A1^+^ macrophages and aneuploid cells play a major role in the cell communication network within the TME (**Figure 12A**). Moreover, there were significant differences in the number and intensity of the signaling networks within the TME between SPP1^-^/SLC11A1^-^ macrophages and SPP1^+^/SLC11A1^+^ macrophages (**Figures 12B, C**). Among these, the TNF signaling pathway was involved in the communication between SPP1^+^/SLC11A1^+^ macrophages and aneuploid cells, and the SEMA3 signaling pathway mediated the communication between aneuploid cells and epithelial cells, endothelial cells, SPP1^+^/SCL11A1^+^ macrophages, and fibroblasts (**Figures 12D, E**). Additionally, the receptor-ligand communication pattern analysis demonstrated that SPP1^-^/SLC11A1^-^ and SPP1^+^/SLC11A1^+^ macrophages had distinct signaling networks, which could be an underlying reason for their differential roles in regulating CRC development (**Figure 12F**). In summary, the findings highlighted the differences in signaling networks between SPP1^-^/SLC11A1^-^ and SPP1^+^/SLC11A1^+^ macrophages in the TME. The communication between macrophages and aneuploid cells offers valuable insight into potential therapeutic targets for CRC.

### Olaparib repositioned as a newly therapeutic candidate against CRC

**Figure 13:**
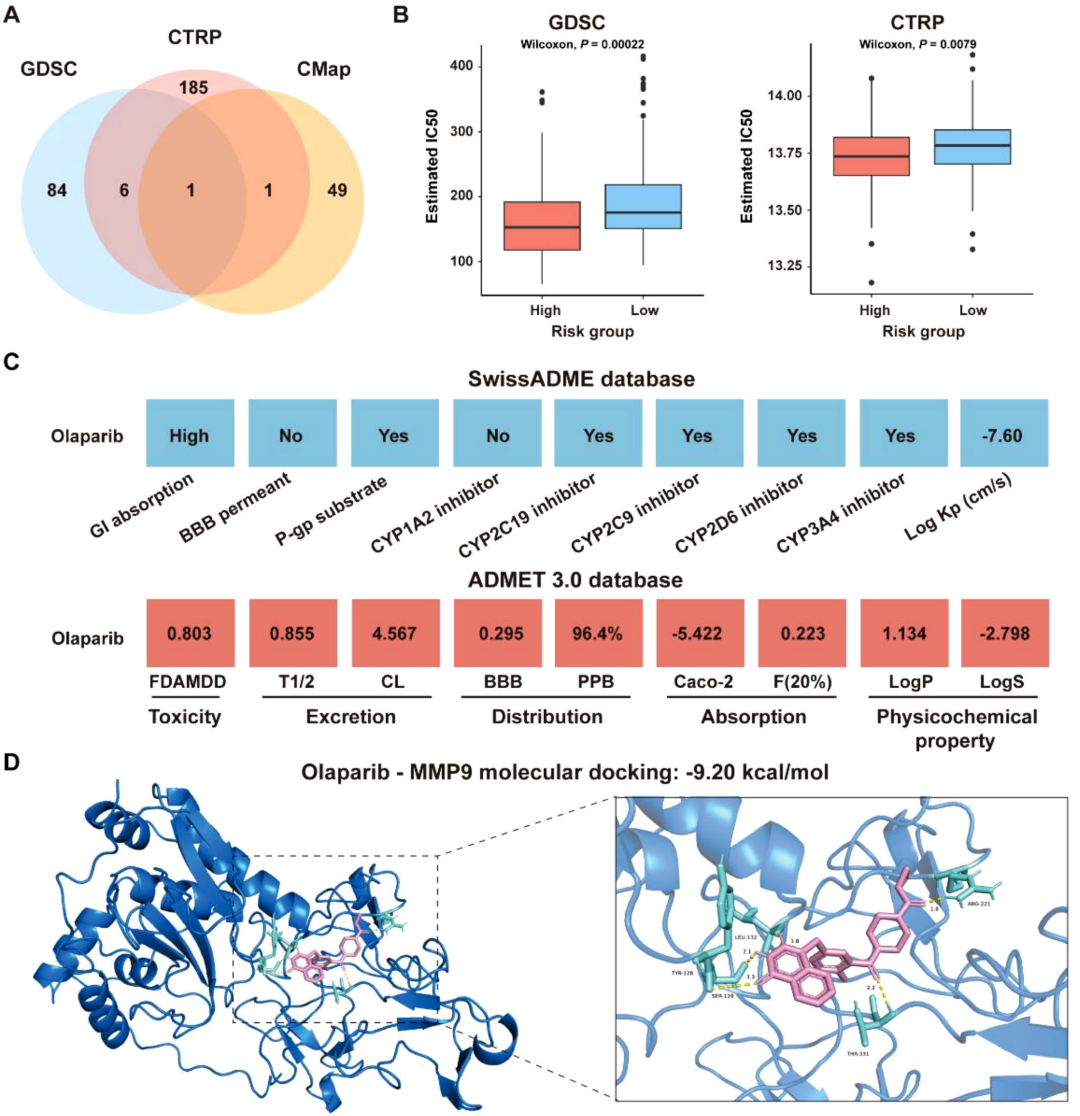
Identification of potential drugs for CRC patients in the GSE17538 dataset. **(A)** Venn diagram showing the number of identified drugs that overlap among the GDSC, CTRP, and CMap databases. **(B)** IC50 values of Olaparib from the GDSC and CTRP databases were compared between the high- and low-risk groups. **(C)** ADMET property analysis of Olaparib based on the SwissADME database and ADMET 3.0 database. **(D)** Molecular docking of Olaparib with MMP-9.

To explore the role of the ICDRS in guiding drug repositioning for CRC, drugs with significant difference in IC50 values between the high-risk and low-risk groups were screened. Based on the GDSC and CTRP databases, 91 and 193 drugs, respectively, were identified with significantly different IC50 values between high- and low-risk groups (*P*<0.01). Additionally, 66 candidate drugs were identified from the CMap database based on the DEGs between the high-risk and low-risk groups in the GSE17538 dataset. Notably, Olaparib was ultimately identified as a common candidate across all three databases through intersection analysis (**Figure 13A**). In both the GDSC and CTRP databases, the IC50 values for Olaparib were significantly higher in the high-risk group than in the low-risk group (GDSC: *P*=0.00022; CTRP: *P*=0.0079 (**Figure 13B**). Additionally, correlation analysis showed that the IC50 values of Olaparib in both the GDSC and CTRP databases were significantly negatively correlated with the expression levels of ICDRS-related genes, including PLA1A, MFNG, LY6E, IFI30, and ADAM8 (*P*<0.05) (**Supplementary Figure S5A**). The ICDRS-derived risk score also showed a negative correlation with the IC50 values of Olaparib in both databases (**Supplementary Figure S5B**). **Supplementary Figure S5C** shows the score of the Olaparib identified from the CMap database. Additionally, the mechanism of action analysis revealed that Olaparib is a PARP inhibitor, providing new insights into the drug’s therapeutic mechanism (**Supplementary Figure S5D**). Furthermore, Olaparib possesses favorable ADMET properties (**Figure 13C**), supporting its potential as a drug for CRC.

To identify the potential targets of Olaparib in CRC, a protein-protein interaction (PPI) network consisting of 337 nodes and 2,751 edges was constructed based on the 346 DEGs between the high-risk and low-risk groups in the GSE17538 dataset (**Supplementary Figure S6A**). The hub genes were identified as the top 5% genes with highest degrees, including BGN, CCL2, COL1A2, COL3A1, CXCL8, DCN, FN1, ITGAM, LUM, MMP-9, POSTN, PTPRC, TGFB1, and THBS1 (**Supplementary Figures S6B, C**). Molecular docking analysis revealed that the binding energies of Olaparib with MMP9, CCL2 and CXCL8 were -9.20 kcal/mol, -7.89 kcal/mol, and -6.05 kcal/mol, respectively (**Figure 13D**, **Supplementary Figures S6D, E**). Overall, these findings indicate that Olaparib could be a promising treatment for CRC. As the high-risk group had a lower IC50 for Olaparib than the low-risk group, indicating that Olaparib could be more beneficial for high-risk CRC patients.

## Discussion

### Development of a novel prognostic signature by integrating ICD-related genes for CRC stratification and personalized therapy

Numerous studies have demonstrated the critical role of ICD-related genes in remodeling the tumor immune microenvironment (TIME), activating the immune system, and influencing therapeutic responses in cancer. For example, Xu et al. (35) discovered that AIM2, a gene related to ICD, could inhibit the growth and metastasis of colon cancer cells *in vivo*, and colorectal adenocarcinoma patients with high expression of AIM2 had an enhanced response to immunotherapy. Li et al. (6) found that ICD could activate TIME to improve the immunotherapy efficiency of cancer patients. Yu et al. (36) identified ICD-related subtypes of CRC characterized by distinct prognostic outcomes, immune landscapes, and somatic mutation profiles, and further developed a prognostic risk signature based on the expression of six ICD-related genes. While these findings underscore the prognostic relevance of ICD in CRC, most existing studies have been limited to bulk RNA-seq data and lack integration with multi-omics data.

To overcome these limitations, our study introduces several key methodological innovations. First, we constructed a prognostic signature based specifically on ICD-related genes, a direction that remains underexplored in CRC. Second, by integrating bulk and single-cell RNA sequencing data, we not only developed a robust signature but also explored key cell types within the TME. Third, unlike most existing machine learning-based models that employ a limited set of algorithms, we comprehensively applied 101 machine learning algorithm combinations and identified the optimal pairing of StepCox (forward) and RSF for signature construction, which demonstrated strong prognostic performance across multiple independent cohorts (**Figure 4A**). Notably, when compared with 60 previously published CRC prognostic models, our ICDRS demonstrated superior predictive accuracy across multiple independent cohorts (**Supplementary Figure S3**). Furthermore, the signature-based nomogram achieved AUC values exceeding 0.9 when predicting 1-year, 2-year, and 3-year OS of CRC (**Figure 6C**). Functional analysis revealed that the signature-derived risk score was significantly correlated with the infiltration level of various immune cells within the TME, including macrophages, immature dendritic cells, and regulatory T cells (**Figure 7B**). Due to the higher likelihood for immune evasion in the high-risk group, low-risk patients were found to be more suitable for immunotherapy (**Figures 8A–J**). Therefore, this study highlights the significance of ICD-related genes in CRC prognosis and provide a comprehensive tool for its prediction.

### The role of SPP1^+^/SLC11A1^+^ macrophages in the TME of CRC

The heterogeneity of macrophages in CRC has gained increasing recognition, with distinct subtypes playing diverse roles in tumor progression and patient outcomes. For example, CD68^+^ and CD163^+^ macrophages have been demonstrated to exert different effects on the prognosis of stages I-III colon cancer patients, highlighting the importance of characterizing macrophage subtypes in CRC (37). In the present study, we found that macrophages exhibited the highest level of ICD activity within the TME of CRC. Notably, two of the 11 ICD-related genes that constitute the prognostic signature, SPP1 and SLC11A1, were exclusively expressed in macrophages within tumor samples (**Figure 11B**). SPP1, also known as osteopontin (OPN), encodes an extracellular matrix protein associated with poor prognosis of CRC patients (38, 39). Similarly, SLC11A1, referred to as natural resistance-associated macrophage protein 1 (NRAMP1), is associated with poor prognosis and resistance to immunotherapy in CRC, and serves as a valuable biomarker for predicting OS of CRC patients (40, 41). Accordingly, a new subtype of macrophages, designated as SPP1^+^/SLC11A1^+^ macrophages, was first identified in this study. Furthermore, a significant difference in the infiltration levels was observed between SPP1^+^/SCL11A1^+^ macrophages and SPP1^-^/SCL11A1^-^ macrophages, with the former exhibiting higher infiltration levels (**Figure 11H**). In addition, the TNF signaling pathway was involved in the communication between SPP1^+^/SLC11A1^+^ macrophages and aneuploid cells (**Figure 12D**). Wan et al. (42) discovered that the secretion of cytokines, such as TNF-α, by macrophages of experimental mice promote epithelial-to-mesenchymal transition (EMT) and CRC progression. This suggests that SPP1^+^/SLC11A1^+^ macrophages may contribute to EMT processes in aneuploid cells, thereby promoting the development of CRC. These findings revealed distinct roles of SPP1^+^/SLC11A1^+^ and SPP1^-^/SLC11A1^-^ macrophages in CRC, with SPP1^+^/SLC11A1^+^ macrophages emerging as a potential new therapeutic target for CRC treatment.

### Olaparib holds the potential as a therapeutic candidate for CRC

With an increasing understanding of the mechanisms underlying ICD, several potential drug candidates have been developed to treat CRC by stimulating ICD. For example, Mao et al. (43) designed a self-assembled nanodrug capable of inducing ICD and reshaping the immunosuppressive TME, to enhance anti-tumor immunity and inhibit CRC progression. Wang et al. (44) demonstrated that Oxaliplatin, a chemotherapeutic agent, could effectively inhibit CRC cell proliferation, promote the release of ICD marker molecules, enhance tumor immunogenicity, and may improve the efficacy of immunotherapies for CRC. Currently, drug repurposing offers a cost-effective and time-efficient strategy to identify new therapeutic applications for existing drugs, accelerating the development of treatments for cancer. Tran et al. (45) discovered that the antipsychotic drug thioridazine can enhance anti-tumor effects in CRC by promoting the release of ICD markers. Olaparib, the first-in-class Poly ADP-ribose polymerase (PARP) inhibitors, has been approved by the FDA for the treatment of ovarian, breast, pancreatic, and prostate cancers (46). Increasing evidence suggests that PARP inhibitors can induce DNA damage responses that lead to ICD, characterized by the release of DAMPs that promote the recruitment and activation of antigen-presenting cells (47). For example, Chen et al. (48) demonstrated that Olaparib enhances radiosensitivity in hepatocellular carcinoma by inducing DNA double-strand breaks, which in turn activate the cGAS-STING pathway. This activation promotes ICD and reshapes the TIME, thereby promoting the antitumor efficacy of radiotherapy in combination with ICIs. Although direct evidence linking Olaparib to ICD induction in CRC remains limited, these findings provide a strong rationale for its potential application in CRC. In this study, Olaparib was identified as a potential drug candidate for treating CRC. Moreover, MMP-9, CCL2, and CXCL8 were identified as potential targets of Olaparib among the hub genes in the PPI network constructed by DEGs between high- and low-risk groups (**Supplementary Figure S6B**). Molecular docking analysis showed that Olaparib had high binding affinity to MMP-9, CCL2, and CXCL8, respectively (**Figure 13D**; **Supplementary Figures S6D, E**). As reported, PARP inhibitors could reduce the expression of MMP-9 (49) and CXCL8 (50), while PARP-1 was able to promote the production of CCL2, thereby activating the CCL2-CCR2 axis (51). Therefore, Olaparib hold promise as a therapeutic agent for CRC by inhibiting the production of these cytokines.

Despite the progress made in elucidating the characteristics of the TME in CRC and identifying a new therapeutic agent, this study still faces several limitations. First, the bioinformatic analysis primarily relies on public datasets, which may not fully capture the complete complexity of the TME. Particularly, the lack of spatial transcriptomic data limits our understanding of the spatial distribution of cells within CRC tissues. Second, while our computational analyses suggest a potential therapeutic role for Olaparib in CRC, direct experimental evidence linking it to the induction of ICD in CRC is currently lacking. To establish its clinical relevance in CRC, *in vitro* studies are essential to assess Olaparib’s effects on CRC cell viability, apoptosis, induction of ICD, and activation of key pathways. Moreover, *in vivo* validation using appropriate CRC animal models is crucial to evaluate the drug’s efficacy in modulating the TIME, enhancing anti-tumor immunity, and improving responses to immunotherapy. Additionally, the prognostic value of the ICDRS and the functional role of key immune cell subsets, such as SPP1^+^/SLC11A1^+^ macrophages, need to be verified through *in vitro* and *in vivo* experiments. Future research should focus on expanding sample sizes, incorporating multi-omics approaches, and leveraging spatial transcriptomics to provide a more comprehensive understanding of CRC pathogenesis and treatment opportunities.

## Conclusion

Using an integrated machine learning framework, a novel ICD-related prognostic signature, termed ICDRS, was developed and applied with robust predictive performance for 1-, 2- and 3-year OS of CRC patients in the training and validation datasets. In particular, ICDRS showed a close association with the infiltration level of immune cell types within the TME. Moreover, a new SPP1^+^/SLC11A1^+^ macrophage subtype, was discovered based on SPP1 and SLC11A1 genes in ICDRS, characterized by high infiltration level. In addition, Olaparib was repositioned as a novel therapeutic candidate for CRC. Overall, the findings of this study highlight the potential of ICDRS as a promising tool for personalized management on CRC prognosis and therapeutics, and deep pathogenic and pharmacological verifications using multi-center and large-sample clinical data would be conducted for further translational applications.

## Data Availability

The original contributions presented in the study are included in the article/**Supplementary Material**, further inquiries can be directed to the corresponding author/s.

## Supplementary material

Supplementary Figure 1 Clustering and annotation of single cells in the GSE132465 dataset. **(A)** The top three marker genes for each cell cluster. **(B)** The top three most highly expressed genes in each cell cluster. **(C)** Bar graph showing the proportion of different cell types in normal and tumor samples. **(D)** t-SNE plot showing ICD activity scores across seven cell types.

Supplementary Figure 2 Multivariate Cox analyses of the risk scores and other clinicopathological factors for OS in the GSE17538, GSE17537, GSE17536, GSE29621, and TCGA-CRC datasets, as well as for DFS in the GSE38832 dataset.

Supplementary Figure 3 Comparison of C-index values of the ICDRS and sixty reported CRC prognostic models in the training and validation sets. ^*^ *P*<0.05, ^**^ *P*<0.01, ^***^ *P*<0.001.

Supplementary Figure 4 Identification of aneuploid cells in the GSE132465 dataset. **(A)** tSNE plot showing the distribution of diploid and aneuploid cells. **(B)** Heatmap illustrating the changes in chromosome CNV across seven cell types. **(C)** tSNE plot showing the division of aneuploid cells into four subpopulations. **(D)** The differentiation trajectory of aneuploid cells. **(E)** Dynamic changes of BST2, LY6E and PLCG2 levels during aneuploid cell differentiation.

Supplementary Figure 5 Analysis of the characteristics of Olanzapine based on the GSE17538 dataset. **(A)** The correlation between the expression levels of the 11 genes comprising ICDRS and the IC50 values of Olaparib in the GDSC databases (left) and CTRP databases (right). **(B)** The correlation between the ICDRS-derived risk score and the IC50 values of Olaparib in the GDSC and CTRP databases. **(C)** The drug repositioning score of Olaparib in the CMap database. **(D)** The mechanism of action of potential drugs, with a focus on Olaparib, identified in the CMap database.

Supplementary Figure 6 Identification of potential targets of Olaparib in the GSE17538 dataset. **(A)** Volcano plot showing the DEGs between the high- and low-risk groups. **(B)** The PPI network constructed from DEGs that identified between high- and low-risk groups. **(C)** The top 14 hub genes in the PPI network. **(D-E)** Molecular docking of Olaparib with CCL2 **(D)** and CXCL8 **(E)**.

## Glossary

CRC: Colorectal cancer
ICD: Immunogenic cell death
TME: Tumor microenvironment
ICDRS: ICD-related signature
OS: Overall survival
DAMPs: Damage-associated molecular patterns
ICB: Immune checkpoint blockade
scRNA-Seq: single-cell RNA sequencing
GEO: Gene Expression Omnibus database
TCGA: The Cancer Genome Atlas
TPM: Transcripts per kilobase million
DFS: Disease-free survival
DEG: Differentially expressed gene
WGCNA: Weighted gene co-expression network analysis
TOM: Topological overlap matrix
ME: Module eigengene
GO: Gene Ontology
KEGG: Kyoto Encyclopedia of Genes and Genomes
BH: Benjamini-Hochberg
HR: Hazard ratio
Enet: Elastic Net
GBM: Gradient Boosting Machine
LASSO: Least absolute shrinkage and selection operator
PlsRcox: Partial least squares regression for Cox
RSF: Random survival forest
SuperPC: Supervised principal components
Survival-SVM: Survival support vector machine
ROC: Receiver operating curve
AUC: Area under the curve
DCA: Decision curve analysis
TMB: Tumor mutational burden
MAF: Mutation annotation format
CNV: Copy number variation
IPS: Immunophenoscore
TIDE: Tumor immune dysfunction and exclusion
CAF: Cancer-associated fibroblast
GDCS: Genomics of drug sensitivity in cancer
CTRP: Cancer therapeutics response portal
CMap: Connectivity map
IC50: Half-maximal inhibitory concentration
PPI: Protein-protein interaction
FBS: Fetal bovine serum
OD: Optical density
GSEA: Gene set enrichment analysis
GSVA: Gene set variation analysis
GS: Gene significance
MM: Module membership
CTLA-4: Cytotoxic T lymphocyte antigen-4
PD-1: Programmed cell death protein 1
ICI: Immune checkpoint inhibitor
TIME: Tumor immune microenvironment
OPN: Osteopontin
NRAMP1: Natural resistance-associated macrophage protein 1
EMT: Epithelial-to-mesenchymal transition
PARP: Poly ADP-ribose polymerase
