# Supplementary material for: Integrated multi-omics and machine learning reveal an immunogenic cell death-related signature for prognostic stratification and therapeutic optimization in colorectal cancer
Source: Front Immunol. 2025 Jul 16;16:1606874. doi: 10.3389/fimmu.2025.1606874 (PMC12307400; doi:10.3389/fimmu.2025.1606874)
Supplement: Supplementary Figure 1 — Clustering and annotation of single cells in the GSE132465 dataset. (A) The top three marker genes for each cell cluster. (B) The top three most highly expressed genes in each cell cluster. (C) Bar graph showing the proportion of different cell types in normal and tumor samples. (D) t-SNE plot showing ICD activity scores across seven cell types. [file DataSheet1.docx]

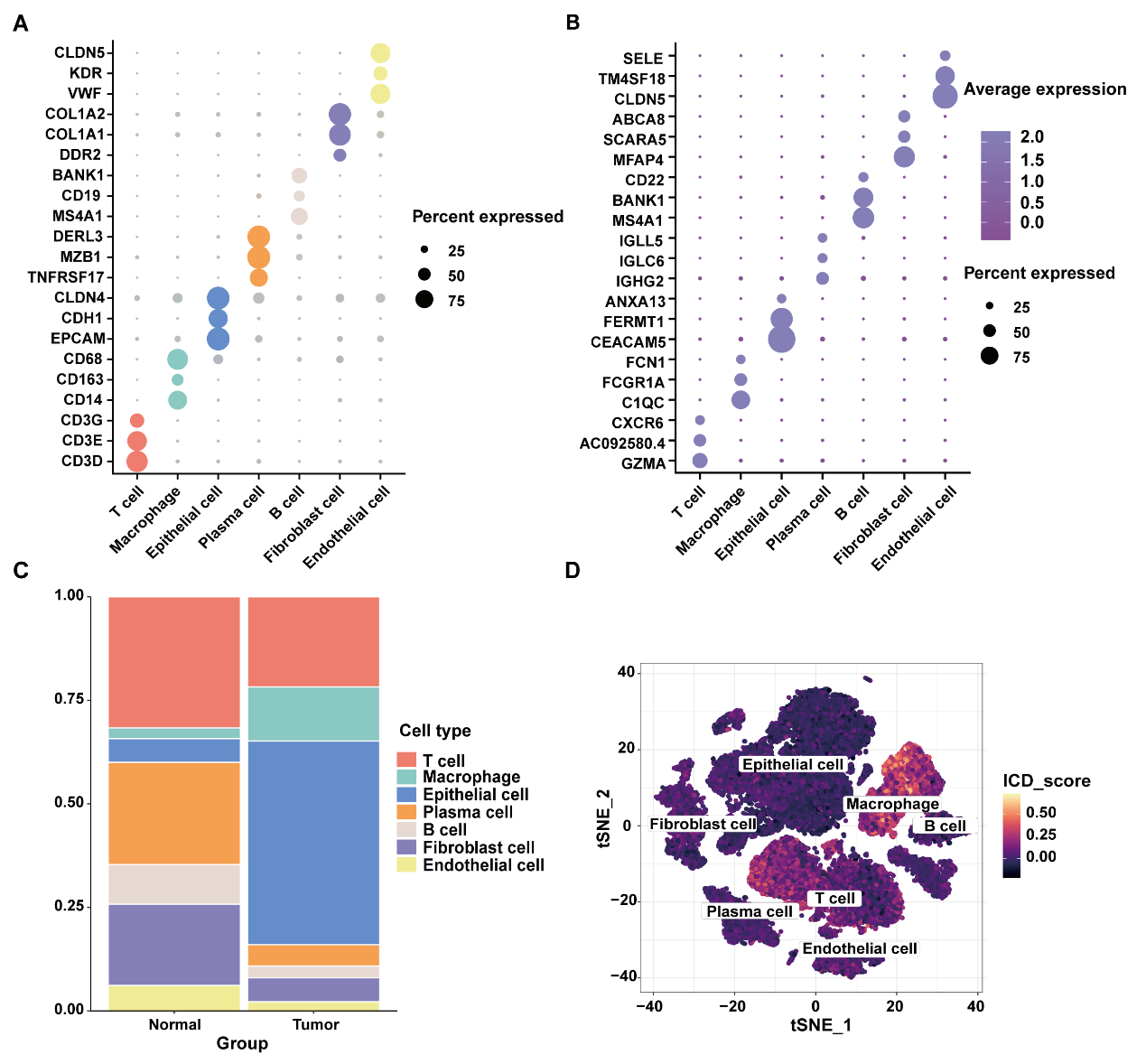


**FIGURE S1** Clustering and annotation of single cells in the GSE132465 dataset. (A) The top three marker genes for each cell cluster. (B) The top three most highly expressed genes in each cell cluster. (C) Bar graph showing the proportion of different cell types in normal and tumor samples. (D) t-SNE plot showing ICD activity scores across seven cell types.


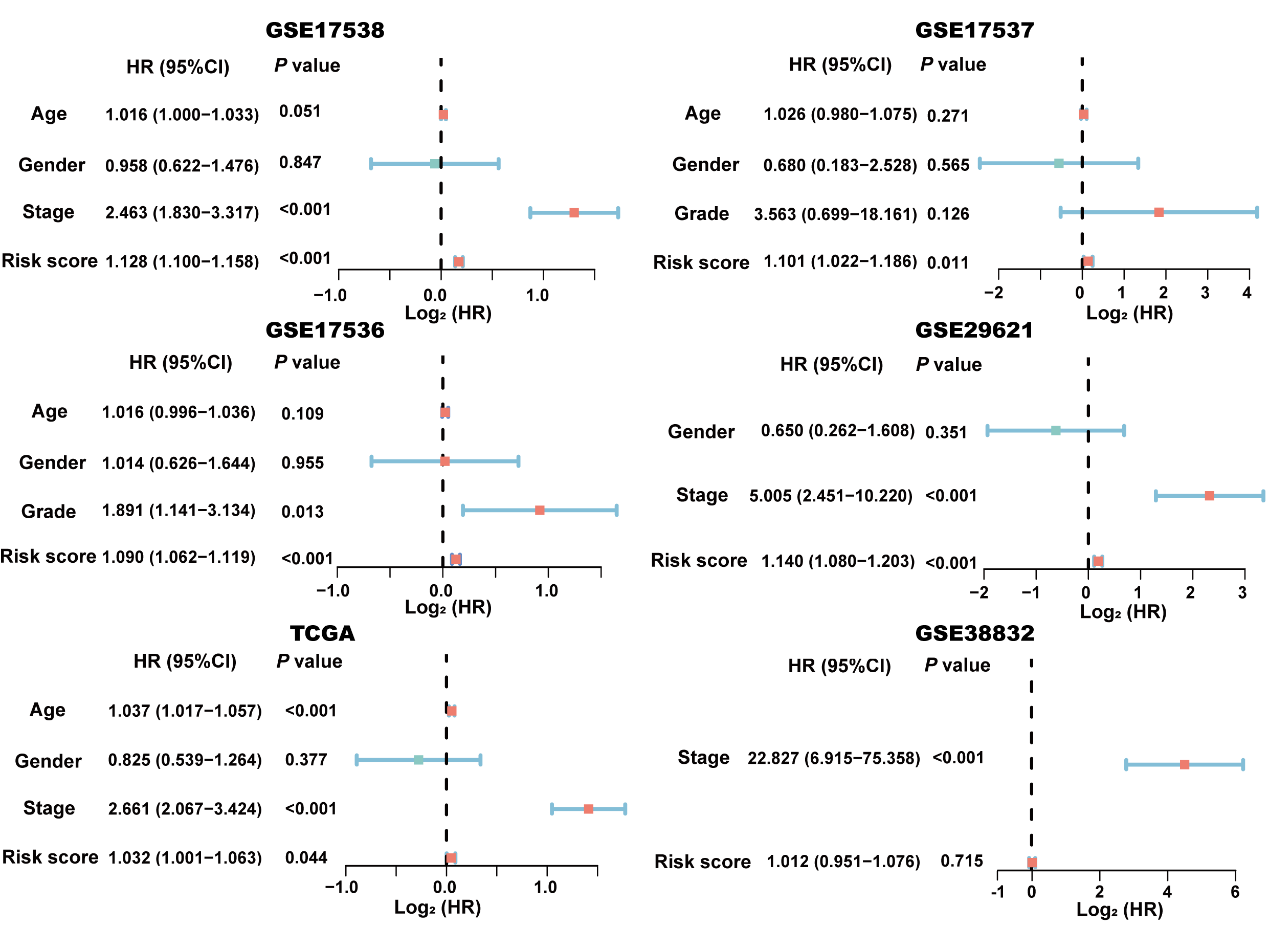


**FIGURE S2** Multivariate Cox analyses of the risk scores and other clinicopathological factors for OS in the GSE17538, GSE17537, GSE17536, GSE29621, and TCGA-CRC datasets, as well as for DFS in the GSE38832 dataset.


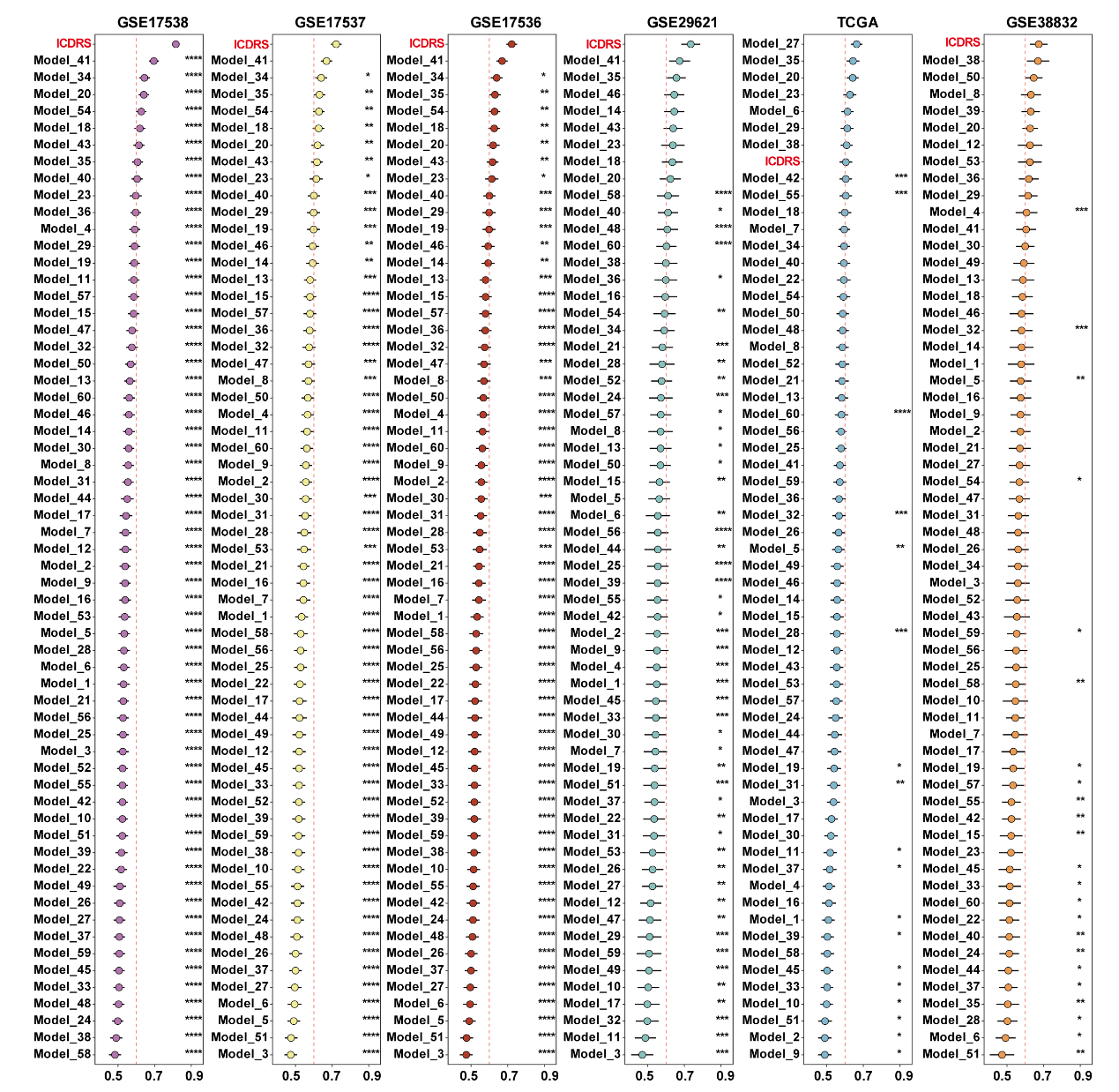


**FIGURE S3** Comparison of C-index values of the ICDRS and sixty reported CRC prognostic models in the training and validation sets. ^*^*P*<0.05, ^**^*P*<0.01, ^***^*P*<0.001.


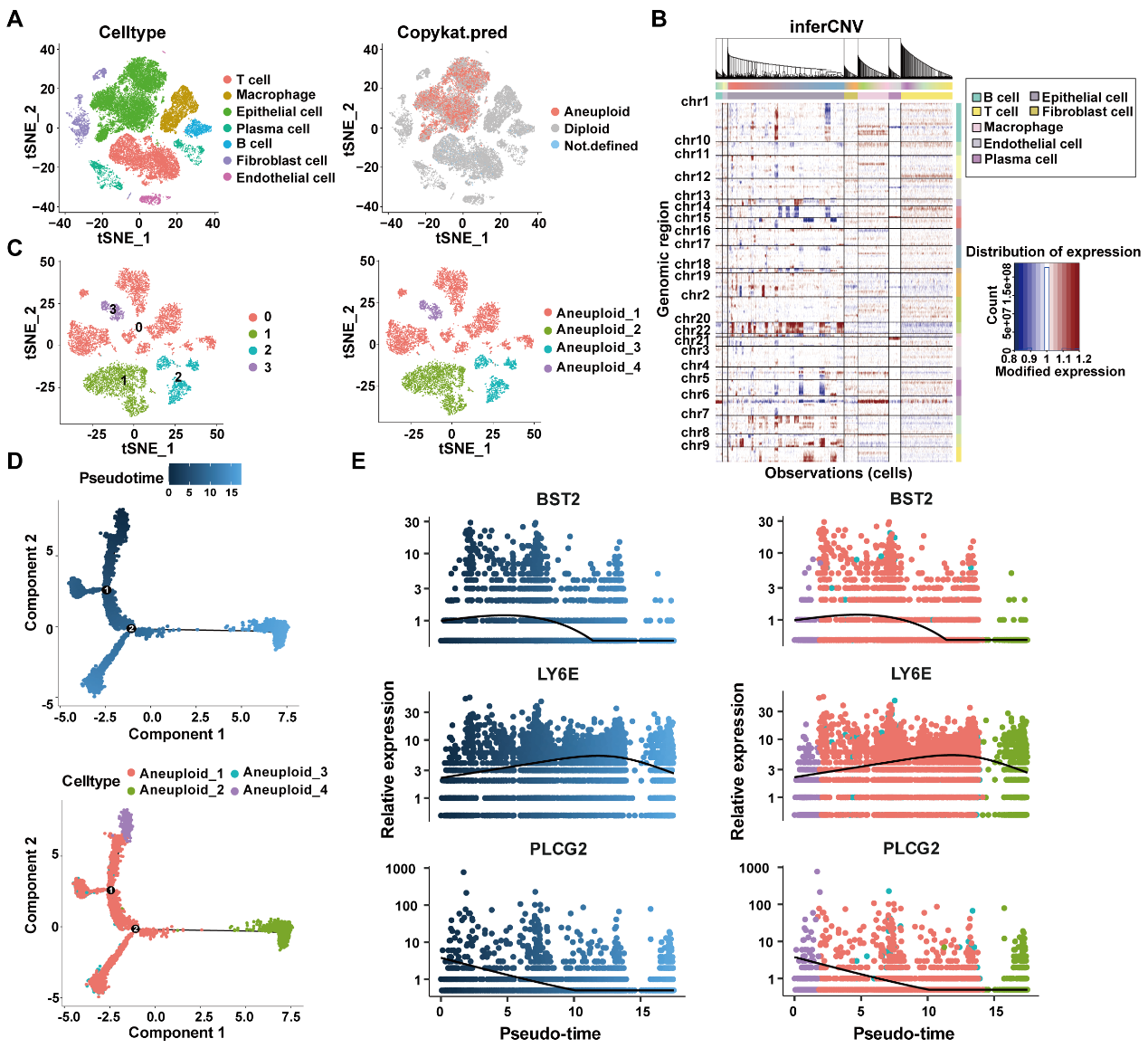


**FIGURE S4** Identification of aneuploid cells in the GSE132465 dataset. (A) tSNE plot showing the distribution of diploid and aneuploid cells. (B) Heatmap illustrating the changes in chromosome CNV across seven cell types. (C) tSNE plot showing the division of aneuploid cells into four subpopulations. (D) The differentiation trajectory of aneuploid cells. (E) Dynamic changes of BST2, LY6E and PLCG2 levels during aneuploid cell differentiation.


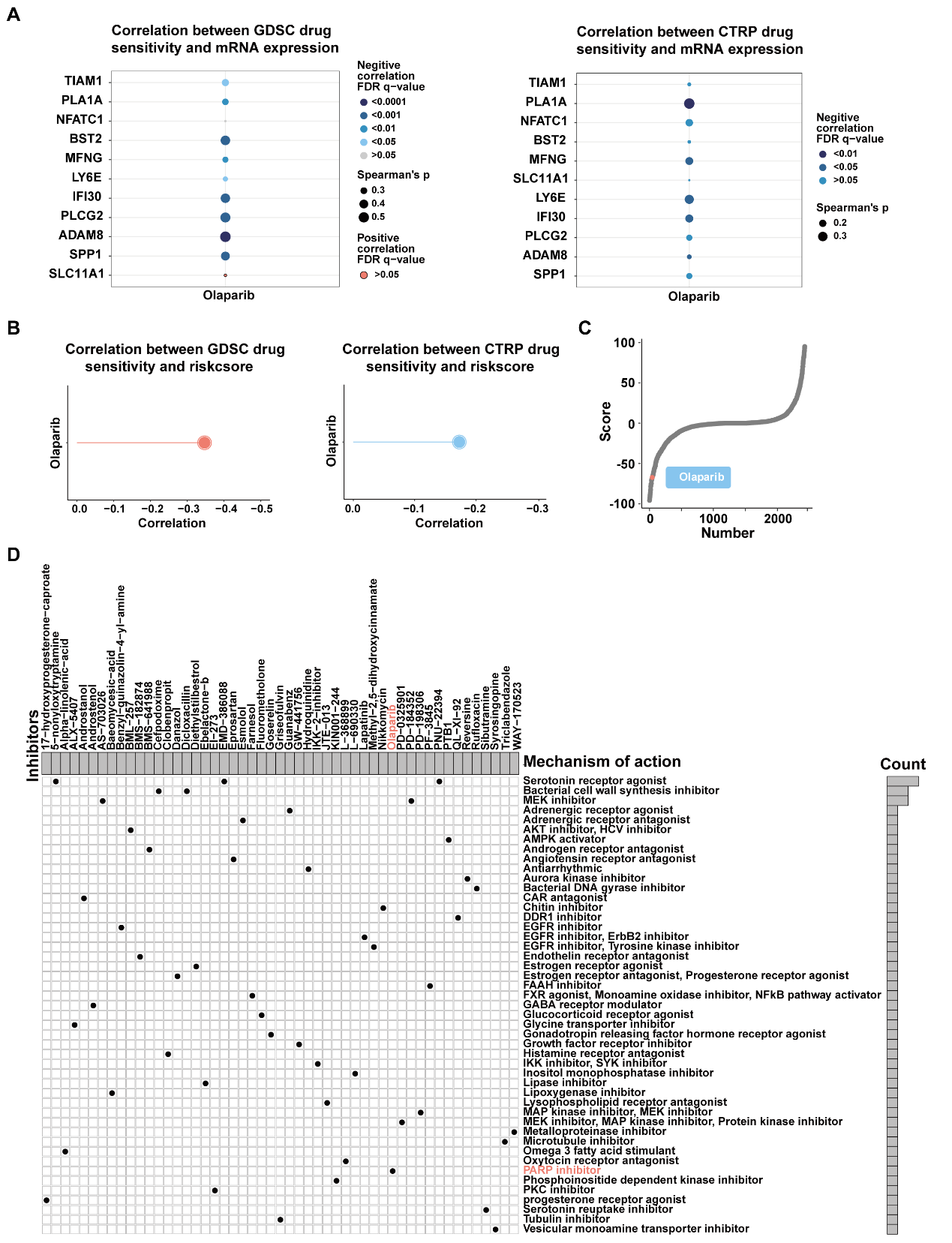


**FIGURE S5** Analysis of the characteristics of Olanzapine based on the GSE17538 dataset. (A) The correlation between the expression levels of the 11 genes comprising ICDRS and the IC50 values of Olaparib in the GDSC databases (left) and CTRP databases (right). (B) The correlation between the ICDRS-derived risk score and the IC50 values of Olaparib in the GDSC and CTRP databases. (C) The drug repositioning score of Olaparib in the CMap database. (D) The mechanism of action of potential drugs, with a focus on Olaparib, identified in the CMap database.


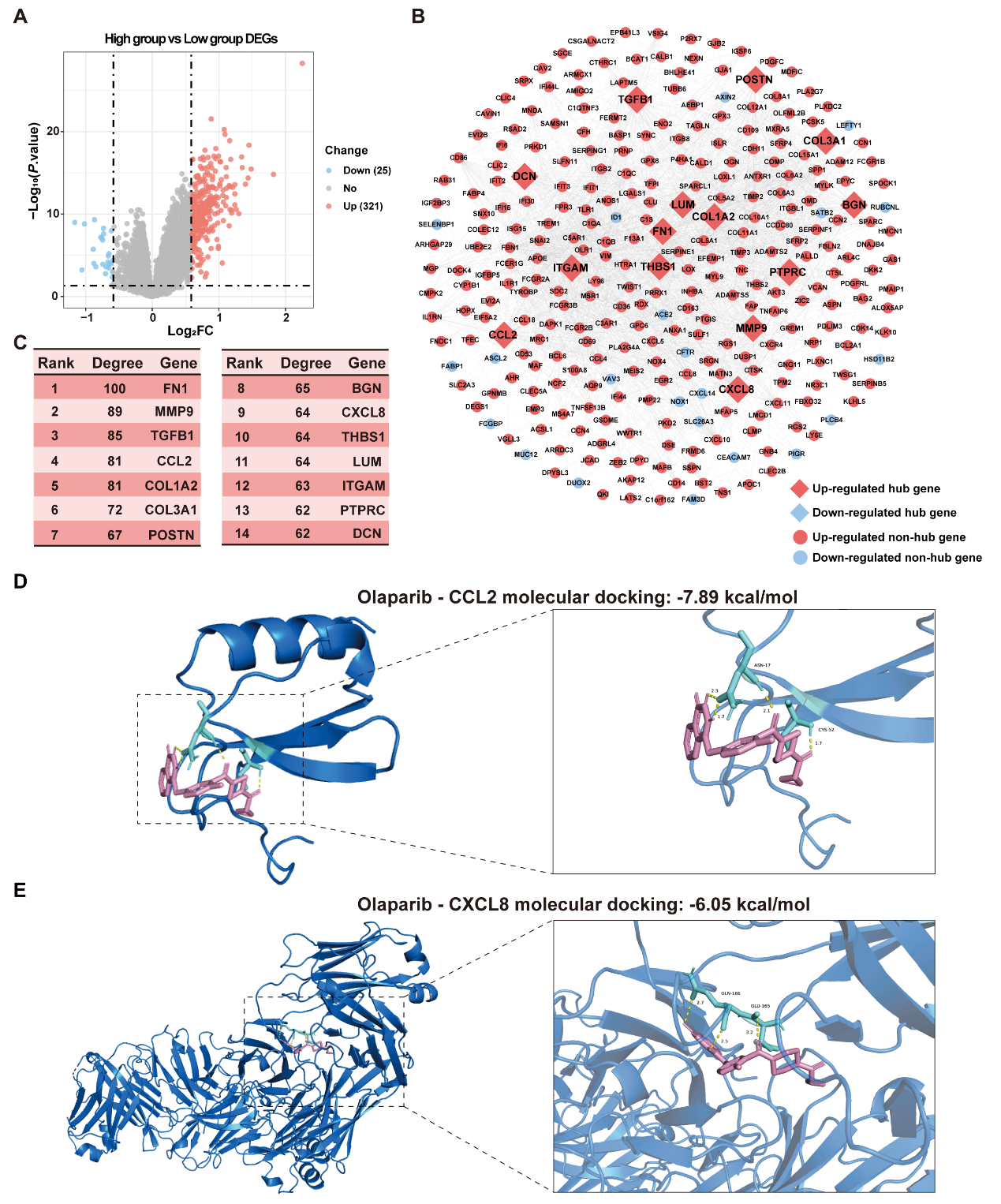


**FIGURE S6** Identification of potential targets of Olaparib in the GSE17538 dataset. (A) Volcano plot showing the DEGs between the high- and low-risk groups. (B) The PPI network constructed from DEGs that identified between high- and low-risk groups. (C) The top 14 hub genes in the PPI network. (D-E) Molecular docking of Olaparib with CCL2 (D) and CXCL8 (E).
